# CD36 mediates SARS-CoV-2-envelope-protein-induced platelet activation and thrombosis

**DOI:** 10.1038/s41467-023-40824-7

**Published:** 2023-08-21

**Authors:** Zihan Tang, Yanyan Xu, Yun Tan, Hui Shi, Peipei Jin, Yunqi Li, Jialin Teng, Honglei Liu, Haoyu Pan, Qiongyi Hu, Xiaobing Cheng, Junna Ye, Yutong Su, Yue Sun, Jianfen Meng, Zhuochao Zhou, Huihui Chi, Xuefeng Wang, Junling Liu, Yong Lu, Feng Liu, Jing Dai, Chengde Yang, Saijuan Chen, Tingting Liu

**Affiliations:** 1 Department of Rheumatology and Immunology, Ruijin Hospital, Shanghai Jiao Tong University School of Medicine, No. 197 Ruijin Second Road, Shanghai, 200025 China; 2 Department of Biochemistry and Molecular Cell Biology, Shanghai Jiao Tong University School of Medicine, 280 South Chongqing Road, Shanghai, 200025 China; 3 Shanghai Institute of Hematology, State Key Laboratory of Medical Genomics, National Research Center for Translational Medicine at Shanghai, Ruijin Hospital, Shanghai Jiao Tong University School of Medicine, Shanghai, 200025 China; 4 Department of Laboratory Medicine, Ruijin Hospital, Shanghai Jiao Tong University School of Medicine, Shanghai, 200025 China; 5 Department of Radiology, Ruijin Hospital, Shanghai Jiao Tong University School of Medicine, Shanghai, 200025 China

**Keywords:** Mechanisms of disease, Research data, Viral infection

## Abstract

Aberrant coagulation and thrombosis are associated with severe COVID-19 post-SARS-CoV-2 infection, yet the underlying mechanism remains obscure. Here we show that serum levels of SARS-CoV-2 envelope (E) protein are associated with coagulation disorders of COVID-19 patients, and intravenous administration of the E protein is able to potentiate thrombosis in mice. Through protein pull-down and mass spectrometry, we find that CD36, a transmembrane glycoprotein, directly binds with E protein and mediates hyperactivation of human and mouse platelets through the p38 MAPK-NF-κB signaling pathway. Conversely, the pharmacological blockade of CD36 or p38 notably attenuates human platelet activation induced by the E protein. Similarly, the genetic deficiency of *CD36*, as well as the pharmacological inhibition of p38 in mice, significantly diminishes E protein-induced platelet activation and thrombotic events. Together, our study reveals a critical role for the CD36-p38 axis in E protein-induced platelet hyperactivity, which could serve as an actionable target for developing therapies against aberrant thrombotic events related to the severity and mortality of COVID-19.

## Abstract (web summary)

Aberrant coagulation and thrombosis are associated with severe SARS-CoV-2 infection. Here, the authors show that the E protein are associated with coagulation disorders in COVID-19 patients and could directly enhance platelet activation and thrombosis through a CD36/p38 MAPK/NF-kB signaling axis.

## Introduction

The coronavirus disease 2019 (COVID-19) pandemic, caused by severe acute respiratory syndrome coronavirus 2 (SARS-CoV-2), has led to a global health crisis^1, 2^. Clinical manifestations range from the common cold to severe pneumonia, multiorgan failure and death^3^. Notably, aberrant coagulation and intensive thrombotic events are frequently observed in COVID-19 patients and reported to be life-threatening complications^4–6^. Indeed, elevated D-dimer and fibrin degradation product (FDP) levels, longer prothrombin time (PT) and activated partial thromboplastin time (APTT) are commonly observed in severe/critical patients and associated with high rate of disseminated intravascular coagulation (DIC)^7–9^; thrombosis of small and mid-sized pulmonary arteries is considered a possible lethal cause of COVID-19 in autopsies studies;^10^ pulmonary embolism (PE) and deep vein thrombosis (DVT) account for a majority of thromboembolic events in severe/critical COVID-19;^11^ local micro-thrombosis resulting from coagulation activation during the disease onset of COVID-19 may lead to disorders in cardiovascular, respiratory, gastrointestinal, and neurological systems, even after the viral clearance^12,13^. In addition, emerging evidence suggests that thrombosis might also be a main cause of long COVID-19 and the risk of DVT and PE is significantly increased in patients post-recovery from COVID-19^14^.

Consistent with the observed thrombotic abnormalities, heightened platelet activation is frequently detected in COVID-19. Platelet activation in COVID-19 was suggested to be involved in immune-thrombosis, which is generally characterized by thrombosis-related aberrant immune responses of complement factors, inflammatory cytokines, immunoglobulins, and the activation of endothelial cells^15,16^. However, recent studies also observed elevated D-dimers and INR in mild/moderate patients without abnormal immune response^17^, implying an alternative mechanism of thrombosis in COVID-19, such as a direct effect of viral-platelet interplay. In fact, the alteration of the transcriptome in platelet post-SARS-CoV-2 infection differs from that in other viral diseases (such as dengue virus and influenza virus), even though thrombosis is also found in these viral illnesses^18,19^. Mechanistically, transcriptome analysis of platelet has demonstrated a role for MAPK signaling in activating the platelets of COVID-19^20^, and the Spike protein has been reported to enhance platelet aggregation induced by various agonists in vitro, although there is still controversy about whether human platelets express angiotensin-converting enzyme 2 (ACE2), the receptor of Spike protein^20,21^. Further research has revealed that toll-like receptors 4 (TLR4), CD42b, and CD147 are alternative receptors for the Spike protein^22–25^. Nevertheless, the role of CD147 in Spike-mediated platelet activation remains controversial as other studies have been unable to observe the binding of the Spike protein to them^26^.

The Envelope (E) protein is the smallest, and perhaps the most enigmatic, major structural protein of SARS-CoV-2, sharing a highly conserved sequence with the E proteins of SARS-CoV and Middle East respiratory syndrome (MERS)-CoV^27,28^. According to recent studies, E protein contributes to COVID-19 progression besides its role in viral assembly and budding. For example, SARS-CoV-2 E protein alone can cause acute respiratory distress syndrome (ARDS)-like damages, inducing rapid cell death and robust secretion of cytokines^29^, whereas SARS-CoV-2 with inactivation or absence of the E protein exhibits decreased titers and attenuated virulence^30,31^. The E protein has also been identified as an independent virulence factor with ion channel activity^32^, and it can be sensed by TLR2 to promote inflammatory cytokine production before the viral entry^33^, highlighting its essential role in COVID-19 pathogenesis. Nevertheless, the pro-thrombotic ability of E protein remains to be explored.

Here, we showed the presence of E protein in the serum of a significant fraction of COVID-19 patients and we demonstrated that the E protein potentiated thrombus formation in vivo in murine models. In vitro, the E protein could directly interact with CD36 on the cell surface of the platelets, leading to activation of the platelets via the p38-MAPK signaling pathway. Last, we demonstrated that the enhancing effect of E protein on both platelet activation and thrombosis was abrogated by *CD36* null mutation or pharmacological blockade of CD36.

## Results

### Serum levels of the SARS-CoV-2 E protein correlate with thrombosis in COVID-19 patients

**Fig. 1:**
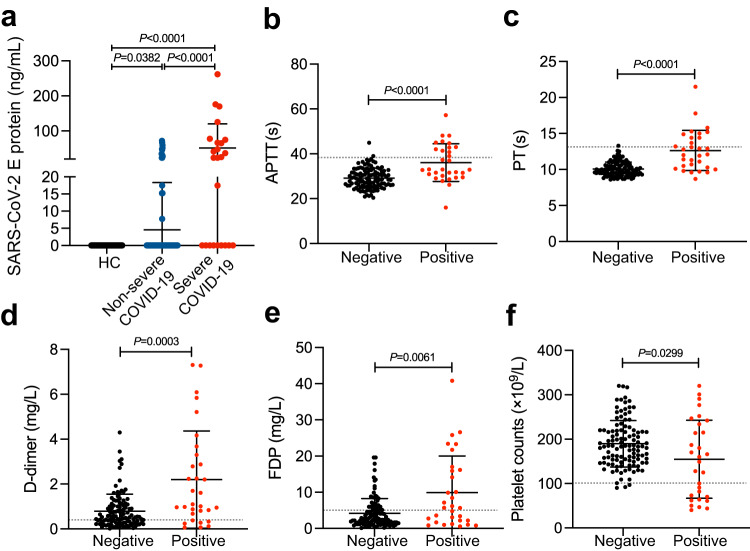
Serum levels of the SARS-CoV-2 E protein correlate with thrombosis in COVID-19 patients. **a** The E protein was detected in 31 COVID-19 patients. The level of the E protein in COVID-19 patients was 12.33 ± 34.87 ng/ml. The COVID-19 patients were divided into a non-severe group (*n* = 121) and a severe group (*n* = 24) as described in the “Methods” section. The level of the E protein was significantly increased in sera of severe COVID-19 patients (51.43 ± 68.63 ng/ml) compared to non-severe COVID-19 patients (4.58 ± 13.77 ng/ml). Significantly longer activated partial thromboplastin time (APTT) (**b**) and prothrombin time (PT) (**c**), elevated D-dimer (**d**) and fibrinogen degradation products (FDP) (**e**), decreased platelet counts (**f**) were observed in COVID-19 patients with positive E test (*n* = 31), compared to COVID-19 patients with negative E test (*n* = 114). The gray line represents the cut-off level. Data are presented as mean ± SD. Data were analyzed by the Kruskal–Wallis test (**a**) or 2-tailed Mann-Whitney *U* test (**b**–**f**). Source data are provided as a Source Data file.

To evaluate the relationship between the serum levels of SARS-CoV-2 E protein and thrombotic events in COVID-19, we used an in-house direct ELISA assay to measure E protein in sera of 145 COVID-19 patients at the time of admission to the hospital (24 patients showed severe symptoms and 121 showed non-severe symptoms during hospitalization; see Methods for details of patient categorization). While no trace amount of E proteins was detected in the sera of 51 age-and sex-matched healthy donors (negative controls), notable levels of the E protein were detected in 31 out of the 145 (21.4%) COVID-19 sera samples (mean: 57.7 ng/mL: ranges: 7.8-261.7 ng/mL), and the serum E protein levels were significantly higher in severe patients than in non-severe patients (Fig. 1a).

Although none of the COVID-19 patients presented with thrombosis upon hospital admission, 12 developed thrombotic events, of whom 7 were severe COVID-19 and 5 were non-severe, within the duration of hospitalization (mean: 18.3 days: ranges: 15–26 days). Notably, the serum levels of E protein positively correlated with diagnostic coagulation parameters such as elevations in activated partial thromboplastin time (APTT), prothrombin time (PT), increased D-dimer and fibrinogen degradation products (FDP) (Fig. 1b–e and Supplementary Fig. 1a–d). Moreover, the serum levels of E protein negatively correlated with platelet counts (Fig. 1f and Supplementary Fig. 1e), though there was a significant positive correlation between the levels of CD62P in plasma, which serves as a marker for platelet activation (Supplementary Fig. 1f). The odds ratio for thrombosis with serum E levels was 1.015 (1.004–1.027) (Table 1), suggesting that E protein is a significant indicator of the thrombosis of COVID-19.

**Table 1:** Relative risks of serum E levels for thrombosis and abnormal coagulant parameters

Thrombosis and abnormal parameters	No. of events (%)	Odds ratio (95%CI) for serum E levels and events	*P* value
Thrombosis	12 (8.3)	1.015 (1.004–1.027)	0.009
APTT Prolongation	14 (9.7)	1.038 (1.018–1.059)	<0.001
PT Prolongation	13 (8.7)	1.045 (1.022–1.069)	<0.001
Elevated D-dimer	78 (53.8)	1.028 (1.006–1.051)	0.014
Elevated FDP	47 (32.4)	1.008 (0.998–1.019)	0.113
Thrombocytopenia	16 (11.0)	1.016 (1.004–1.028)	0.007

Logistical regression analysis was performed to calculate the odds ratios for serum E levels and events.

*APTT* activated partial thromboplastin time, *PT* prothrombin time, *FDP* fibrinogen degradation products.

### Intravenous administration of E protein promotes thrombosis in mice

**Fig. 2:**
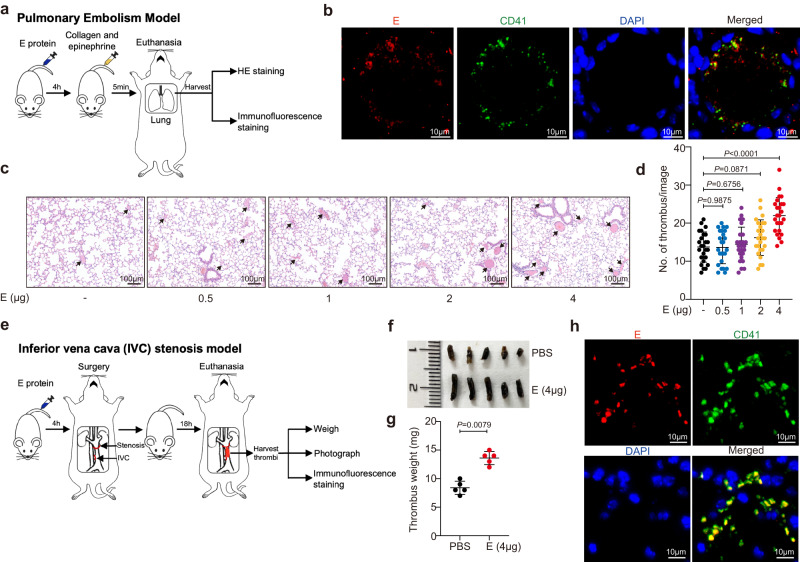
Serum E proteins promote thrombosis in vivo. **a** Wild-type (WT) mice were intravenously injected with the E protein (0.5, 1, 2 and 4 μg per mouse) or PBS. Four hours later, mouse models of pulmonary embolism (PE) were induced by intravenous injection of collagen and epinephrine. **b** The presence of the E protein on platelets in lung embolism of mice. Immunofluorescence staining on lung section from the E-treated mouse model of PE was performed with anti-SARS-CoV-2 E protein (red), and anti-CD41 (green) antibodies. Nuclei were stained with 4′,6-diamidino-2-phenylindole (DAPI). Scale bar = 10 μm. **c** Representative field on hematoxylin-eosin (HE) stained lung sections in each group. Scale bar = 100 μm. Results in **b** and **c** were confirmed in five independent experiments. **d** Quantification of the number of lung embolisms per visual field on lung sections. The number of lung thrombi was 13.72 ± 3.96 in PBS-treated WT mice, 13.64 ± 4.28 in E-treated (0.5 μg) WT mice, 14.44 ± 4.51 in E-treated (1 μg) WT mice, 16.20 ± 4.67 in E-treated (2 μg) WT mice, and 22.00 ± 4.74 in E-treated (4 μg) WT mice. Data are mean ± SD of 25 counts from 5 mice in each group. **e** WT mice were treated with the E protein (4 μg per mouse) or PBS, and then the inferior vena cava (IVC) stenosis model was performed. **f** Representative image of the thrombi isolated from mouse IVC. **g** The thrombus weight was 8.40 ± 1.14 mg in PBS-treated mice, and 13.60 ± 1.14 mg in the E-treated mice (*n* = 5). Data are presented as mean ± SD. **h** Immunofluorescence staining of the E protein (red), CD41(green), DAPI in thrombi section isolated from the E-treated mouse after IVC model. Scale bar = 10 μm. Data were analyzed by the Kruskal–Wallis test (**d**) or 2-tailed Mann–Whitney *U* test (**g**). Source data are provided as a Source Data file.

Given the presence of E protein in COVID-19 patient blood, we hypothesized that circulatory E proteins might be pro-thrombotic in vivo. To test this, we established a mouse model of pulmonary embolism, whereby thrombi in pulmonary vessels were induced by a mixture of collagen and epinephrine upon intravenous administration of the E protein (Fig. 2a)^34^. We injected 0.5, 1, 2, or 4 μg of E protein per mouse in these models and found that only the highest dose (4 μg of E protein per mouse) resulted in a robust increase of thrombi in pulmonary vessels (Fig. 2c, d). In such cases, immunofluorescence staining revealed elevated attachment of E protein to platelets, marked by CD41, at the thrombi site of pulmonary vessels (Fig. 2b and Supplementary Fig. 2). These results provided evidence that E protein can cause pulmonary embolism when present above a certain threshold level in the blood. According to the dose translation between laboratory animals and humans in drug development^35^, 4 μg of E protein per mouse is estimated to be equivalent to 240 ng/ml in the sera of humans, which was within the upper range of serum E proteins in patients (261.7 ng/mL; Fig. 1a).

Because of the high prevalence of deep venous thrombosis in severely or critically ill COVID-19 patients^6, 14^, we further evaluated the effect of E protein in a surgery-induced inferior vena cava (IVC) stenosis model (Fig. 2e). The average thrombus weight in mice injected with the E protein (4 μg/mice) was significantly increased as compared to that of the PBS control group (Fig. 2f, g). Increased attachment of E protein to platelets was also confirmed by immunofluorescence stain in the thrombi isolated from the IVC of mice (Fig. 2h).

### E protein induces platelet activation via the p38 MAPK/NF-kB signaling pathway

**Fig. 3:**
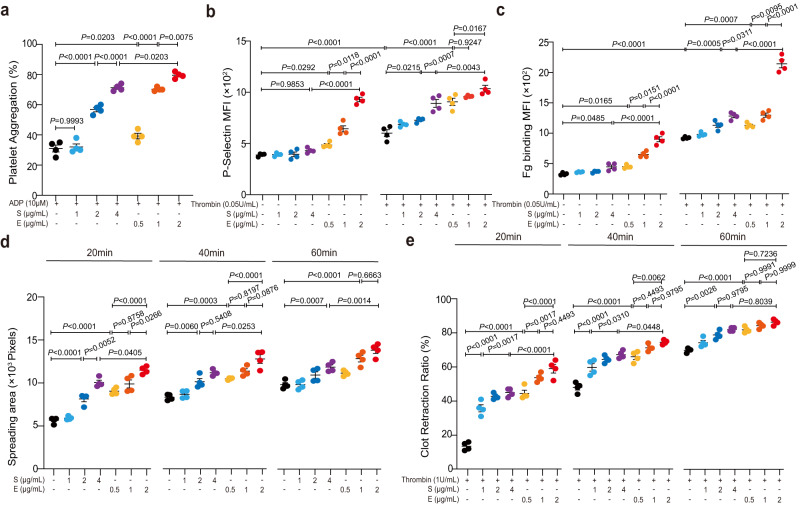
E protein activates platelets. Washed human platelets from healthy donors were incubated with the SARS-CoV-2 E protein (0.5, 1, 2 μg/ml) or the SARS-CoV-2 S protein (1, 2, 4 μg/ml) for 5 min at 37 °C as a pretreatment step. **a** Aggregation of pretreated platelets was measured in response to ADP (10 μM) (*n* = 4). Both the E protein and the S protein enhanced platelet aggregation in a dose-dependent manner. The E protein exhibited a stronger ability to enhance platelet aggregation than S protein. **b** and **c** The E protein dose-dependently promoted platelet P-selectin exposure and integrin αIIbβ3 activation. P-selectin exposure and fibrinogen (Fg) binding in pretreated platelets with or without stimulation of 0.05U/ml thrombin were detected by flow cytometry (*n* = 4). **d** Pretreated platelets were allowed to spread on immobilized Fg at 37 °C for 20, 40, and 60 min. Area (pixel numbers) of 3 random fields of spreading platelets were quantified (*n* = 4). **e** The E protein concentration-dependently accelerated clot retraction. Pretreated platelets were added into platelet-free plasma, and then clot retraction was induced by 1 U/ml thrombin (*n* = 4). Quantification of clot retraction at 20, 40, and 60 min, respectively. Data were analyzed by 1-way ANOVA with Tukey multiple-comparisons test (**a**–**c**) or 2-way ANOVA with Tukey multiple-comparisons test (**d** and **e**). Data are presented as mean ± SEM. Source data are provided as a Source Data file.

To investigate the mechanism underlying E protein’s pro-thrombotic effect, we turned to examine how the E protein interacted with platelets isolated from healthy people in vitro. Since S protein was previously found to promote platelet activation phenotype^24,25^, it was used in these experiments as a positive control. We found that, like S protein, E protein could act in a dose-dependent manner to enhance ADP-dependent platelet aggregation (Fig. 3a and Supplementary Fig. 3a–d), P-selectin exposure (with or without thrombin; Fig. 3b and Supplementary Fig. 3e), fibrinogen (Fg) binding (Fig. 3c and Supplementary Fig. 3e), platelet spreading (Fig. 3d and Supplementary Fig. 3f) and clot retraction (Fig. 3e and Supplementary Fig. 3g). In these assays, E protein appeared to show an even greater potential to potentiate platelet activation. For example, 2 μg/ml of E protein induced more significant increase in platelet aggregation and P-selectin exposure than 4 μg/ml of S protein (Fig. 3a, b). On the other hand, when incubated with endothelial cells, E protein did not seem to cause a change in the expression of ICAM-1 and VCAM-1 (Supplementary Fig. 4a–c), an observation that might suggest that the E protein did not notably activate endothelial cells, though further studies are required to address the impact of E protein on the function of endothelial cells.

**Fig. 4:**
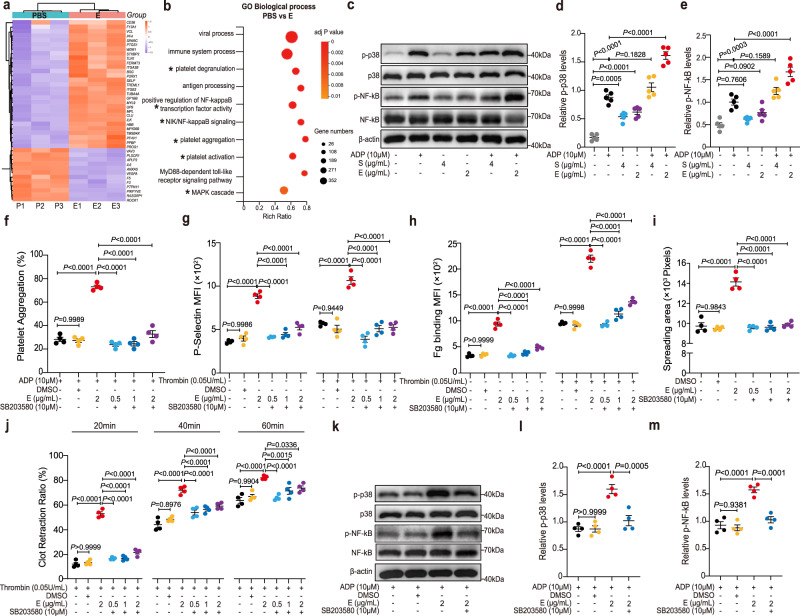
The p38 MAPK/NF-*κ*B pathway mediates E protein-induced platelet activation. **a** Washed human platelets isolated from healthy donors were incubated with or without the E protein (2 μg/ml) at 37 °C, and then platelet RNA was isolated for RNA-Seq (*n* = 3). Heat map of significantly differentially expressed platelet transcripts from human platelets treated with or without the E protein. **b** Gene Ontology (GO) analysis identified enriched pathways (Log_2_FC ≥ 1, adjusted *P* < 0.05). **c**–**e** Human platelets were preincubated with the E protein (2 μg/ml) or the S protein (4 μg/ml) for 5 min at 37 °C and stimulated with or without ADP (10 μM). Western blot analysis showed that the E protein potentiated the phosphorylation of p38 and NF-*κ*B in platelets (*n* = 5). Washed human platelets were pretreated with 10 μM SB203580 (a p38 inhibitor) or dimethyl sulfoxide (DMSO) for 10 min at 37 °C, followed by incubation with the E protein (0.5, 1, 2 μg/ml) for 5 min as a pretreatment step. **f** Potentiated platelet aggregation induced by the E protein combined with ADP 10 μM was suppressed by SB203580 (*n* = 4). **g** and **h** Enhanced P-selectin exposure and fibrinogen (Fg) binding of platelets induced by the E protein were suppressed by SB203580 (*n* = 4). **i** SB203580 inhibited the E protein promoting human platelet spreading on Fg (*n* = 4). Pretreated platelets were allowed to spread on a Fg-coated surface at 37 °C for 1 h. Data was shown with quantification analysis of the areas of spreading platelets (pixel numbers). **j** SB203580 attenuated accelerating clot retraction induced by the E protein in response to thrombin (*n* = 4). Data were presented with quantification analysis of clot retraction at 20, 40, and 60 min. **k**–**m** Western blot analysis of phosphorylation of p38 and NF-*κ*B in platelets. SB203580 (10 μM) suppressed the elevated phosphorylated levels of p38 and NF-*κ*B promoted by the E protein (2 μg/ml) in response to ADP (10 μM) (*n* = 4). The molecular weight markers are shown (**c**, **k**). Data were analyzed by 1-way ANOVA with Tukey multiple-comparisons test (**d**–**i**, **l**, **m**) or 2-way ANOVA with Tukey multiple-comparisons test (**j**). Data are presented as mean ± SEM. Source data are provided as a Source Data file.

To investigate the intracellular factors mediating SARS-CoV-2 E protein-induced platelet activation, we used E protein- or PBS-treated platelets for RNA-seq experiments. Differentially expressed gene (DEG) analysis revealed that many genes associated with platelet activation were up-regulated by the E protein—for example, *SELP*, *ITGA2B*, *ITGB3* and *CD36* (Fig. 4a). Gene Ontology (GO) analysis confirmed that E-protein-upregulated genes were indeed significantly associated with biological processes (BP) such as platelet activation, aggregation, and degranulation (Fig. 4b).

Based on GO analysis, E protein-up-regulated genes were also associated with the mitogen-activated protein kinase (MAPK) and NF-*κ*B signaling pathways (Fig. 4b), two pathways that were previously suggested to be activated in COVID-19 patients to mediate the cytokine production and drive the inflammation^20–37^. Though these pathways were known to be mediated by a signaling cascade involving various protein kinases including the extracellular signal-regulated kinase (ERK), the c-Jun terminal kinase (JNK) and the p38 group of protein kinase (p38 MAPK)^38^, we found that E protein treatment did not seem to affect the phosphorylation of ERK or JNK (Supplementary Fig. 5a–c), while significantly promoting the phosphorylation of p38 and NF-*κ*B in platelets upon stimulation with low-dose of ADP (Fig. 4c–e). Even in the absence of ADP, a notable increase in the phosphorylated levels of p38 in platelets was observed after E protein treatment (Fig. 4c, d). Consistent with previous studies, COVID-19 platelets exhibited higher reactivity, as characterized by increased aggregation capacity under ADP stimulation (Supplementary Fig. 6a, b), elevated P-selectin exposure (Supplementary Fig. 6c), increased spreading area on immobilized Fg (Supplementary Fig. 6d, e), and enhanced clot contraction (Supplementary Fig. 6f, g). Of note, both the protein expression of CD36 and the phosphorylation of p38 MAPK and NF-*κ*B were significantly upregulated in COVID-19 platelets with or without the presence of agonists (Supplementary Fig. 6h–k).

To test if the p38 MAPK pathway was indeed involved in E protein-induced platelet activation, we treated platelets with both SB203580 (a p38 inhibitor) and E protein. The result showed that SB203580 significantly blocked E protein-induced platelet aggregation upon stimulation with ADP, and this effect was more evident when platelets were treated with a lower concentration of the E protein (Fig. 4f and Supplementary Fig. 7a). Likewise, SB203580 suppressed a variety of E protein-induced platelet activation phenotypes, including P-selectin exposure (Fig. 4g), Fg binding (Fig. 4h), platelet spreading on Fg (Fig. 4i and Supplementary Fig. 7b), clot retraction (Fig. 4j and Supplementary Fig. 7c), and phosphorylation of p38 and NF-*κ*B (Fig. 4k–m). Together, these results indicated that the p38 MAPK pathway mediated E protein-enhanced platelet activation.

### The membrane protein CD36 mediates E protein-induced platelet activation

**Fig. 5:**
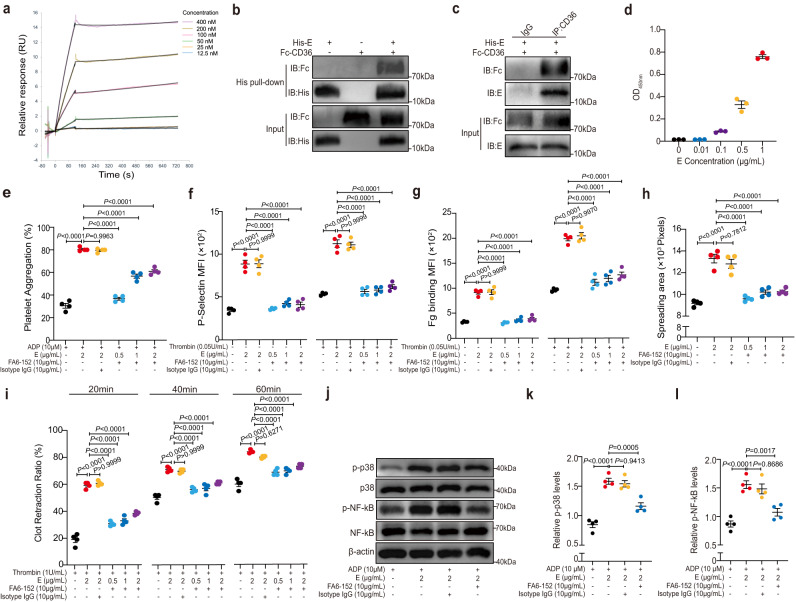
The transmembrane protein CD36 mediates E protein-induced platelet activation. **a** Biacore sensorgrams and binding kinetics determined by SPR for recombinant SARS-CoV-2 E protein with recombinant human CD36 protein. **b** His pull-down assay showed that his tagged E protein could specifically pull down the recombinant CD36. **c** Coimmunoprecipitation showed that recombinant CD36 coimmunoprecipitated with the E protein. Results in (**b** and **c**) were confirmed in three independent experiments. **d** The E protein bound to immobilized CD36 in a dose-dependent manner. **e** FA6-152 (10 μg/ml) ameliorated enhanced platelet aggregation induced by the E protein (*n* = 4). **f** and **g** Potentiated P-selectin exposure and fibrinogen (Fg) binding of platelets induced by the E protein were diminished by FA6-152 (*n* = 4). P-selectin exposure and binding of Fg to platelets were measured with or without stimulation of thrombin (0.05 U/ml). **h** FA6-152 suppressed the E protein-enhanced human platelet spreading on Fg (*n* = 4). Platelets were allowed to spread on immobilized Fg at 37 °C for 1 h. Areas (pixel numbers) of 3 random fields of spreading platelets were quantified (*n* = 4). **i** Accelerated clot retraction in response to thrombin promoted by the E protein was abolished by FA6-152 (*n* = 4). Quantification of clot retraction at 20, 40, and 60 min. **j**–**l** Western blot analysis showed that FA6-152 (10 μg/ml) diminished the increased phosphorylation of p38 and NF-*κ*B promoted by the E protein (2 μg/ml) in response to ADP (10 μM) (*n* = 4). The molecular weight markers are shown (**b**, **c**, **j**). Data were analyzed by 1-way ANOVA with Tukey multiple-comparisons test (**e**–**h**, **k**, **l**) or 2-way ANOVA with Tukey multiple-comparisons test (**i**). Data are presented as mean ± SEM. Source data are provided as a Source Data file.

Platelet activation is typically initiated post the binding of soluble agonists or adhesion molecules on certain receptors on platelets, which then transduces intracellular signaling pathways^39^. In an attempt to screen for the factors that could interact with the E protein and transduce platelet activation signals in platelets, we incubated human platelet lysates with His-tagged E protein immobilized on the HisPur cobalt resin and then subjected the pulled-down proteins for mass spectrometry analysis. A notable membrane protein pulled down by the E protein was CD36 (Supplementary Table 2), a glycoprotein that has been implicated in mediating platelet activation and thrombosis under conditions of hyperlipidemia or inflammation^40,41^. In a surface plasmon resonance experiment, protein-protein interaction between E protein and recombinant human CD36 was further confirmed, and the affinity was measured using the parallel kinetics method. The results showed a high affinity between these two proteins (KD = 55 nM, Fig. 5a). We further confirmed that E protein could directly interact with CD36 by repeating the pull-down assay using His-tagged E protein and a recombinant CD36 (Fig. 5b), as well as co-immunoprecipitation experiments with the recombinant CD36 followed by immunoblotting with anti-E protein antibody (Fig. 5c). A solid-phase ELISA assay additionally showed that the E protein (10–1000 ng/ml) dose-dependently bound with immobilized CD36 (Fig. 5d). Consistently, the colocalization of the E protein and CD36 were identified on platelets isolated from COVID-19 patients (Supplementary Fig. 8).

Next, we used FA6-152, an anti-human CD36 antibody, to block CD36 in platelet activation experiments. Notably, FA6-152 treatment attenuated the ability of E protein to promote platelet aggregation (Fig. 5e and Supplementary Fig. 9a). The anti-CD36 antibody also decreased the effect of E-protein-induced platelet *α*-granule secretion (Fig. 5f), Fg binding (Fig. 5g), platelet spreading on immobilized Fg (Fig. 5h and Supplementary Fig. 9b), clot retraction (Fig. 5i; Supplementary Fig. 9c), as well as E protein-induced elevation of the phosphorylation levels of p38 and NF-*κ*B (Fig. 5j–l). Thus, CD36 was involved in activating the E protein-induced p38 MAPK/NF-*κ*B pathway and platelet activation.

### *CD36* deficiency or blockade attenuates E protein-enhanced thrombosis in mice

**Fig. 6:**
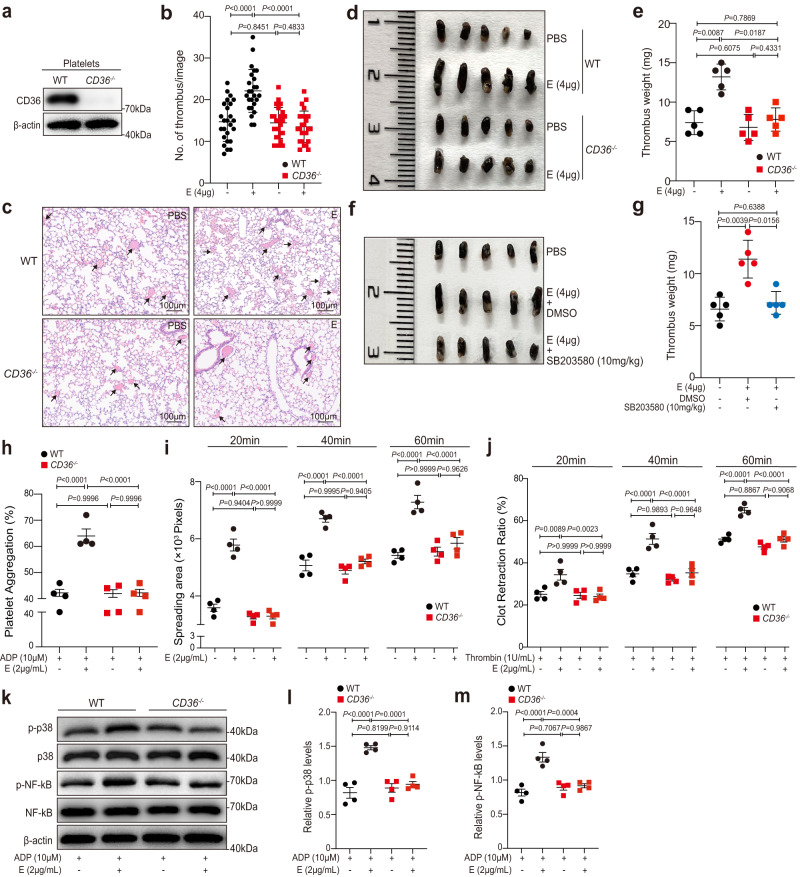
*CD36* deficiency or CD36 blockade in vivo attenuates E protein-enhanced thrombosis. **a** Levels of CD36 in wild-type (WT) and *CD36*^−*/*−^ mouse platelets. Results were confirmed in three independent experiments using platelets from different mice. **b** and **c** WT and *CD36*^−*/*−^ mice were intravenously injected with the E protein (4 μg per mouse) or PBS 4 h before the pulmonary embolism (PE) model. **b** Quantification of the numbers of lung embolisms per visual field on lung sections. Data are mean ± SD of 25 counts from 5 mice in each group. **c** Representative field on lung sections by hematoxylin-eosin (HE) staining from WT and *CD36*^−*/*−^ mice injected with the E protein or PBS. Scale bar = 100 μm. **d** and **e** Inferior vena cava (IVC) stenosis model was performed in WT and *CD36*^−*/*−^ mice. WT and *CD36*^−*/*−^ mice were pretreated with the E protein (4 μg per mouse) or PBS. **d** Representative image of the IVC thrombi. **e** The quantification analysis of the thrombi weight (*n* = 5). **f** and **g** WT mice were intraperitoneally injected with 10 mg/kg SB203580 or DMSO. After 2 h, mice were intravenously infused with the E protein (4 μg per mouse) or PBS, and then the IVC models were applied (*n* = 5). **h** Aggregation enhanced by the E protein was attenuated in *CD36*^−*/*−^ mice platelets in response to ADP (*n* = 4). Platelets from WT and *CD36*^−*/*−^ mice were incubated with the E protein or PBS for 5 min at 37 °C, followed by stimulation with ADP. **i** The promoting effects of the E protein on platelet spreading were abolished in *CD36*^−*/*−^ mouse platelets (*n* = 4). Data were shown with quantification analysis of the areas of spreading platelets (pixel numbers) at 20, 40, and 60 min. **j** Potentiated clot retraction induced by the E protein was suppressed in *CD36*^−*/*−^ mouse platelets (*n* = 4). **k**–**m** The phosphorylation levels of p38 and NF-*κ*B were decreased in E-treated *CD36*^−*/*−^ mouse platelets induced by ADP compared to E-treated WT mouse platelets (*n* = 4). The molecular weight markers are shown (**k**). Data were analyzed by the Kruskal–Wallis test (**b**, **e**, **g**), or 2-way ANOVA with Tukey multiple-comparisons test (**h**–**j**, **l**, **m**). Data are presented as mean ± SD (**b**, **e**, **g**) or mean ± SEM (**h**–**j**, **l**, **m**). Source data are provided as a Source Data file.

The above finding that viral E protein could act through CD36 to potentiate platelet activation suggested the latter as a candidate target for therapeutic intervention to reduce the risk of thrombosis in COVID-19. To assess this possibility, we used the aforementioned mouse model of pulmonary embolism to compare the effect of intravenous administration of 4 μg of E protein in *CD36*^*−/−*^ or WT mice (Fig. 6a; PBS injection was used as a control in both genetic backgrounds). We first confirmed that the peripheral platelet counts in *CD36* deficiency mice were not significantly changed as compared to WT mice (Supplementary Fig. 10a) and reproduced the pro-thrombotic effect of E protein in WT mice (compare Fig. 6 and Fig. 2). But unlike what was observed in WT animals, the number of lung vasculature thrombi was nearly identical between the E protein- and PBS-administrated *CD36*^*−/−*^ mice (Fig. 6b, c). Similarly, when deep venous thrombosis was induced in the IVC mouse model after the administration of E protein, the resultant average thrombus weight in *CD36*^*−/−*^ mice were significantly decreased compared to those in WT mice (Fig. 6d, e). The decrease in thrombus weight in the IVC model was also observed when the p38 inhibitor SB203580 was administered to E protein-injected WT mice (Fig. 6f, g).

We then isolated platelets from WT or *CD36*^*−/−*^ mice and examined the features normally associated with platelet activation. The results showed that *CD36* deficiency abolished various E protein-induced platelet activation phenotypes, including platelet aggregation in response to ADP (Fig. 6h and Supplementary Fig. 10b, e, f), platelet spreading on immobilized Fg (Fig. 6i and Supplementary Fig. 10c), clot retraction (Fig. 6j and Supplementary Fig. 10d), and increased phosphorylation of p38 and NF-*κ*B (Fig. 6k–m). Thus, *CD36* deficiency broadly protected the mice from the E-protein-induced thrombosis phenotype.

## Discussion

Here we have provided multiple lines of evidence to indicate a role for CD36 in SARS-CoV-2 E protein-induced pro-thrombotic state that potentially underlies the coagulation and thrombosis disorders. Our study is based on: (1) surface plasmon resonance, protein pull-down, co-immunoprecipitation, mass spectrometry, and solid-phase ELISA assay to identify and validate the interaction between the E protein and CD36; (2) in vitro platelet activation assay using human and mouse platelets incubated with recombinant E protein to demonstrate the effect of E protein inducing platelet hyperactivity phenotypes; (3) in vivo mouse models of pulmonary embolism and IVC stenosis under the condition of *CD36* deficiency and pharmacological inhibition of anti-CD36 antibody (FA6-152) or p38 inhibitor (SB203580) to demonstrate the CD36 and p38 in the pro-thrombotic state of platelets and forming arterial and venous thrombosis in vivo. Together, these experiments point to a model whereby the viral E protein, presumably as part of the circulatory virions in the blood, directly engages CD36 on the platelet membrane, which leads to activation of the intracellular p38 MAPK/NF-*κ*B pathway and then promotes platelet activation.

Of note, previous studies have shown that the E protein, both as a recombinant soluble protein and as a native membrane protein associated with SARS-CoV-2 viral particles, can physically interact with the TLR2 transmembrane receptor in a specific and dose-dependent manner, and the E-TLR2 interaction can stimulate the NF-κB transcription factor and production of the CXCL8 inflammatory chemokine in cells overexpressing TLR2 (e.g., HEK-TLR2 cell line)^33,37^. Moreover, the viral S protein can independently bind with CD42b, another membrane protein, to stimulate platelets. In these contexts, our finding that CD36 is a receptor for the E protein suggests that SARS-CoV-2 may interact and activate platelets via multiple routes—that is, through S-CD42b and E-CD36—on the surface of platelets, so that the virion may elicit systemic abnormality of the host homeostasis system without entering the platelets. Interestingly, our study indicates that deficiency or blockade of CD36 alone is sufficient to abolish E-protein-induced platelet activation, suggesting that the binding activities (i.e., S-CD42b and E-CD36) were synergistic, rather than redundant. Further biochemical and/or structural studies, however, are necessary to illuminate the mutual interaction between the virion and the platelet.

So far, a variety of other pathologic abnormalities have been observed in critical or fatal cases, including endothelial dysfunction^42^ and NETs formation^43^, which could be potentiated by platelet hyperactivity as a consequence of aggregation of platelets to endothelial cells and neutrophils^20^. Thus, the E protein-induced activation of platelets to the viral E protein may be part of multiple immune cell responses, either in sequence or in parallel, to SARS-CoV-2 infection. Previously, the E protein has been suggested as a virulence factor to activate the monocytes to induce cytokine storm^33^. In the future, it would be of interest to investigate how CD36 signaling participate in these series of immune responses. Furthermore, studies have shown that COVID-19 patients may experience changes in blood lipid levels, including increased levels of triglycerides and low-density lipoprotein cholesterol (LDL-C)^44,45^, and dysregulated platelet lipid metabolism plays a role in the occurrence of thrombotic complications in COVID-19^46^. Considering that CD36 is a key receptor molecule involved in the cellular regulation of lipid influx, future studies are warranted to explore the role of E protein in the regulation of platelet lipid metabolism^38,40^.

In conclusion, our study illustrated a mechanism that the E protein of SARS-CoV-2 could directly enhance platelet activation and thrombosis through a CD36/p38 MAPK/NF-*κ*B signaling axis. Given that COVID-19 is a risk factor for thrombosis and appropriate thromboprophylaxis is still in need to avoid thrombotic events^14^, our study opens a potential avenue for therapeutically counteracting thrombotic complications of COVID-19. Considering that the E protein is relatively conserved among highly pathological coronaviruses^28,47^, targeting the route of E-protein-induced thrombotic events may provide a generally applicable means to ameliorate the pathogenesis of thrombosis post-coronavirus infection.

## Methods

### Patient enrollment

One hundred and forty-five COVID-19 patients were recruited from the isolation ward of Ruijin Hospital (Shanghai, China) between 18 April and 31 May 2022. Fifty-one age- and sex-matched healthy donors were enrolled as controls. All COVID-19 patients met the diagnostic criteria for COVID-19, and SARS-CoV-2 infection was confirmed by reverse transcription polymerase chain reaction (RT-PCR). COVID-19 symptoms were diagnosed as mild (common flu-like symptoms without pneumonia), moderate (mild pneumonia), severe (respiratory frequency ≥30/min, blood oxygen saturation <93% at rest, the ratio of arterial oxygen partial pressure to fractional inspired oxygen [PaO_2_/FiO_2_] <300, and/or pulmonary inflammation progressing >50% within 24 to 48 h), or critical (respiratory failure, septic shock, and/or multiple organ dysfunction)^7^. Subsequently, patients with mild or moderate symptoms were classified into the non-severe group, and those with severe or critical symptoms into the severe group. The characteristics of all participants were summarized in Supplementary Table 1. Blood samples from COVID-19 patients at the time of hospital admission or from healthy donors were collected. After centrifugation of the clotting blood, the serum was separated and kept frozen until use. We enrolled a cohort of 91 COVID-19 patients, all of whom had not undergone antiplatelet drug therapy in the one month prior to enrollment. We obtained serum samples to assess the levels of E protein and plasma samples to measure CD62P levels. All the participants in this study were Asians. In this study, sex was not included as a variable in the study design and analysis due to the absence of conclusive evidence suggesting a sex bias in COVID-19 infection at the time of the study. As a result, the researchers chose a consecutive enrollment approach and did not conduct sex selection when enrolling both COVID-19 patients and healthy controls. Since sex was not a targeted variable in the study, the data related to sex-specific differences were not collected or analyzed separately. All participants were recruited under study protocols approved by the Institutional Review Board of Ruijin Hospital (ID: 2022-71), Shanghai Jiao Tong University School of Medicine. Written informed consent was obtained from all participants.

### Measurement of SARS-CoV-2 E protein in serum

An in-house direct enzyme-linked immunosorbent assay (ELISA) was performed to measure the serum levels of SARS-CoV-2 E protein. Briefly, standard protein (recombinant SARS-CoV-2 E protein, #RP01263, ABclonal Technology) at an initial concentration of 2000 ng/ml was serially doubling diluted in PBS with 1% bovine serum albumin (BSA) to construct a seven-point standard curve. 96-well plates (Costar) were coated with standard protein or serum from patients or healthy donors overnight at room temperature (RT). After washing with Wash Buffer (PBS with 0.05% Tween 20) three times, a Blocking Buffer (PBS with 5% BSA) was added to the wells for 2 h at RT. The plates were then washed three times. Rabbit anti-SARS-CoV-2 Envelope antibody (4 µg/mL, #NBP3-07959, Novus biologicals) was diluted in Blocking Buffer and added into each well for 2 h at RT. After washing three times, diluted goat anti-rabbit horseradish peroxidase (HRP)-conjugated antibodies (1:1000, #7074S, Cell Signaling Technology) were incubated on the plate for 2 h at RT. The plates were washed to remove unbound antibodies and reacted with peroxidase substrate TMB for 20 min at RT. The reaction was stopped by adding a half volume of 1.0 M sulfuric acid. The optical density was measured at 450 nm with a spectrophotometer. E protein concentrations of patient or healthy control sera were determined by comparing against the standard curve. Data were collected by Gen5 CHS software (version 2.09).

### Evaluation of soluble CD62P levels in plasma

The levels of soluble CD62P were measured in plasma from COVID-19 patients using the commercial ELISA kit (#DY137, R&D Systems) according to the manufacturer’s instructions.

### Mice

*CD36* knockout (*CD36*^−*/*−^) on C57BL/6 genetic background and C57BL/6 control mice (both male and female, 6–8 weeks, 20–25 g) were purchased from Cyagen Bioscience, Inc.. All animal procedures were performed in accordance and approved by Shanghai Jiao Tong University School of Medicine. In this study, all animal handling, welfare, monitoring, and euthanasia practices were performed in strict accordance with the ethical guidelines. More specifically, mice were housed in a specific pathogen-free environment on 12-h light/dark cycles with free access to food and water, ambient temperature 22–24 °C, and humidity 50–70%. Mice were handled using tunnels or an open hand approach instead of being picked up by the tail, aiming to minimize stress and promote their well-being. Animals were regularly monitored by qualified personnel to ensure their health and well-being. If necessary, euthanasia was performed using CO_2_ inhalation, following approved protocols. All animal experiments were performed in a randomized and blinded manner. In this study involving a mouse experiment, the consideration of sex as a variable in the study design and analysis was omitted due to the limited availability of evidence suggesting sex-based differences in COVID-19 pathogenesis in mice. As a result, we presented the data in an aggregated manner for sex. Measured values were excluded in case of technical failure during the experiment. At least five mice were used for each experimental group. The numbers of mice for each experiment were shown in the respective Figure.

### Platelet aggregation assay

Human blood was collected from the antecubital vein into a blood collection tube containing buffered sodium citrate. Washed platelets were separated from platelet-rich plasma (PRP) by centrifugation at 1000 × *g* for 10 min and resuspended in Tyrode buffer. Mouse platelets were isolated as described previously^48,49^. Washed platelets were adjusted to approximately 3 × 10^8^ platelets per microliter. Platelets were incubated with SARS-CoV-2 E protein (#RP01263, ABclonal Technology) or S protein (#RP01283LQ, ABclonal Technology) for 5 min at 37 °C before starting each functional assay. P38 inhibitors (SB203580, Selleck Chemicals) or Anti-CD36 antibodies (10 µg/mL, FA6-152, #ab17044, Abcam) were incubated with platelets for 10 min before being treated with the E protein. Aggregation of platelets in response to ADP was measured in a lumi-aggregometer (Chrono-Log, Havertown, PA) under stirring conditions (900 rpm) at 37 °C. Data were recorded by using Aggrolink software (ChronoLog, USA).

### Platelet spreading assay

Analysis of platelet spreading was processed as described^50^. After preincubation, platelets were allowed to adhere to immobilized fibrinogen (20 μg/ml) on glass slides for 20, 40 or 60 min at 37 °C. Spreading platelets were fixed, permeabilized, and stained with rhodamine-conjugated phalloidin and captured with a Zeiss microscope. The platelet area was quantified using the Image J software (v2.9.0, National Institutes of Health, Bethesda, MD).

### Clot retraction

Washed platelets adjusted to approximately 3 × 10^8^ platelets were subjected to preincubation, mixing 100 μl platelets with 300 μl human platelet-poor plasma (PPP). The clot retraction was induced by stimulation of thrombin (1 U/ml) at 37 °C and monitored by taking photographs at indicated time points (20, 40 or 60 min).

### Fibrinogen binding and P-selectin exposure assay

For fibrinogen (Fg) binding and P-selectin exposure assay, pretreated platelets were incubated with PE-conjugated anti-CD62P antibodies (1:100, clone AK-4, #555524, Becton Dickinson Biosciences) and AF647 conjugated Fg (1:50, #F35200, Life Technologies) for 20 min at room temperature and measured by a flow cytometer (FACS CantoII, Becton Dickinson). To determine the platelet purity, washed human platelets were incubated with APC-conjugated anti-CD41 antibodies (1:100, clone HIP8, #559777, Becton Dickinson Biosciences) and FITC-conjugated anti-CD45 antibodies (1:100, clone HI30(RUO), #555482, Becton Dickinson Biosciences) for 20 min at room temperature before measurement. Data collection was performed by BD FACSDiva software (version 8.0.1).

### Platelet RNA sequencing

Platelets isolated from healthy volunteers were incubated with or without SARS-CoV-2 E protein at 37 °C. Platelet RNA was extracted using the miRNeasy Mini Kit (QIAGEN) following the manufacturer’s instructions. Total RNA was qualified and quantified using a Nano Drop and Agilent 2100 Bioanalyzer (Thermo Fisher Scientific, MA, USA). RNA sequencing was performed on the BGISEQ500 platform (BGI-Shenzhen, China) and the sequencing reads were filtered with SOAPnuke (v1.5.2). Clean reads were mapped to the reference genome (genome version # GCF_000001405.39_GRCh38.p13) using HISAT2 (v2.0.4). Differential expression genes (DEGs) analysis was carried out by DESeq2 (v1.4.5) with log_2_foldchange (log_2_FC) > 1.0 and adjusted *P* value < 0.05. GO enrichment analyses were performed online at http://www.geneontology.org/.

### His-tag pull-down assay and mass spectrometry

His pull-down assay was performed using Pierce^TM^ His Protein Interaction Pull-down Kit (#21277, Thermo Scientific^TM^) according to the manufacturer’s instructions with minor modification^51^. Briefly, protein lysate was prepared from platelets isolated from healthy volunteers. His-tagged SARS-CoV-2 E protein was incubated with HisPur cobalt resin for 1 h at 4 °C. After washing, the platelet lysate was added to the resin containing the immobilized His-tagged E protein and incubated at 4 °C for 2 h, after which the binding complex was eluted. The elution samples were subjected to SDS-PAGE for immunoblotting or mass spectrometry (MS).

Gel pieces were excised from SDS-PAGE gels and de-stained. Subsequently, the gel pieces were dehydrated using a vacuum centrifuge. The proteins within the gel were subjected to reduction, followed by alkylation. Next, the gel pieces were subjected to overnight digestion with trypsin at a concentration of 12.5 ng/μl. The resulting peptides were extracted using 60% ACN with 0.1% trifluoroacetic acid (TFA). The extracts were pooled together and then completely dried using a vacuum centrifuge. The peptide mixture was analyzed with a C18-reversed phase analytical column (Thermo ScientificTM EASY Column, 10 cm long, 75 μm inner diameter, 3 μm resin) and a Q Exactive mass spectrometer that was coupled to Easy nLc (Thermo ScientificTM). MS data was acquired using a data-dependent top 20 method, selecting abundant precursor ions from the survey scan (300–1800 m/z) for HCD fragmentation. Parameters included an AGC target of 1e6, maximum inject time of 50 ms, and one scan range. A dynamic exclusion of 30.0 s was applied. The initial files underwent processing followed by database screening with MASCOT engine (Matrix Science, London, UK; version 2.2). For protein identification, the following options were used. Enzyme=Trypsin, Missed cleavage = 2, Fixed modification: Carbamidomethyl (C), Dynamical modifications: oxidation (M), acetyl (K) (protein N-term). In-gel digestion, MS analysis, and database searching were done by Shanghai Applied Protein Technology Company (Shanghai, China)^52^.

The data of the membrane proteins were searched against the UniprotKB Human Reference Proteome database (http://www.uniprot.org/, up to date as of December 10, 2021), and the search results were filtered by peptides ≥ 2 (Supplementary Table 2).

To confirm CD36 protein as the binding protein in the complex pulled down by His-tagged E protein, the recombinant human CD36 protein (10 μg, #CP94, Novoprotein) was incubated with the resin containing the immobilized His-tagged E protein (10 μg, #RP01263, ABclonal Technology) for 2 h at 4 °C and then eluted. The elution samples were analyzed by SDS-PAGE and the presence of CD36 was verified by immunoblotting.

### Surface plasmon resonance (SPR)

SPR experiments were performed on Biacore 8K (GE Healthcare) Instrument at 25 °C using diluted PBS-P buffer (#273923, Cytiva). His-tagged recombinant SARS-CoV-2 E protein was captured on NTA chips (60 s, 10 µl/min, 30 µg/mL). Serial dilutions of recombinant Human CD36 protein were injected, ranging in concentrations from 400 nM to 12.5 nM for parallel kinetics experiments. The surface was regenerated in 350 mM EDTA (60 s, 30 µl/min). Reference-substrated curves were fitted to a 1:1 binding model using Biacore Insight Evaluation Software (v3.0.12).

### Co-immunoprecipitation

The SARS-CoV-2 E protein and recombinant human CD36 protein mixtures were pretreated with protein G-agarose beads for 1 h at 4 °C. After centrifugation, the supernatants were incubated with anti-CD36 antibodies (10 µg/mL, FA6-152, #ab17044, Abcam) or isotype IgG (10 µg/mL, clone G3A1, #5415, Cell Signaling Technology) for 2 h, and then incubated with protein G-agarose beads overnight on a rocker at 4 °C. The beads were then harvested and rinsed 3 times with weak RIPA lysis buffer. Bead-captured E protein or CD36 was detected by immunoblotting.

### Western blot

The resting platelets or aggregated platelets were added to the same volume of lysis buffer containing 1% protease inhibitor mixture (Sigma, St. Louis, MO, USA). Next, the samples were incubated on ice for 20 min. Equivalent amounts of samples were separated by SDS-PAGE, and protein expression was quantified by western blotting with targeted antibodies^53^. Anti-phospho-p38 (1:1000, clone D3F9, #4511, CST), anti-p38 (1:1000, clone D13E1, #8690, CST), anti-phospho-NF-κB p65 (1:1000, clone 93H1, #3033, CST), anti-NF-κB p65 (1:1000, clone D14E12, #8242, CST), anti-phospho-ERK (1:1000, clone D13.14.4E, #4370, CST), anti-ERK (1:1000, clone 137F5, #4695, CST), anti-phospho-JNK (1:1000, clone 81E11, #4668, CST), anti-JNK (1:1000, clone 56G8, #9258, CST), anti-ICAM-1 (1:1000, #4915, CST), anti-VCAM-1(1:1000, clone E1E8X, #13662, CST), anti-human CD36 (1 µg/mL, #MAB1955, R&D Systems), anti-mouse CD36 (1 µg/mL, SMφ, #sc-7309, Santa Cruz), anti-His-Tag (1:2000, clone AMC0149, #AE003, ABclonal Technology), anti-Human IgG Fc (HRP)(1:3000, #ab97225, Abcam), and biotin-conjugated anti-SARS-CoV-2 envelope (1 µg/mL, #ab284658, Abcam) antibodies were used as primary antibodies. Anti-β-actin antibody (1:2000, #AF5003, Beyotime Biotechnology) was used as an internal control. HRP-conjugated anti-rabbit IgG (1:5000, #7074 S, CST), HRP-labeled anti-Mouse IgG (1:1000, #A0216, Beyotime Biotechnology), and HRP-linked anti-rat IgG (1:3000, #7077, CST) were used as secondary antibodies. Data were collected by GeneSys software (v1.8.6.0) and analyzed by Image J software (v2.9.0).

### Interaction of E protein with CD36 in a solid phase assay

A solid-phase assay was performed as described^37^. Briefly, soluble recombinant CD36 (100 μl at 1 μg/ml, #CP94, Novoprotein) in bicarbonate buffer (PH = 9.6) was coated in 96-well plates. After saturation, the E protein (10-1000 ng/ml, #RP01263, ABclonal Technology) was added for 2 h at 37 °C. CD36-E complex was incubated with rabbit anti-SARS-CoV-2 Envelope antibody (4 µg/mL, #NBP3-07959, Novus biologicals) followed by goat anti-rabbit HRP-conjugated antibodies (1:1000, #7074 S, Cell Signaling Technology). After reacting with TMB and stopping the reaction, the optical density was measured at 450 nm with a spectrophotometer.

### Pulmonary embolism model

A pulmonary embolism model was processed as described^34^. Age- and sex-matched, 6- to 8-week-old WT mice and *CD36*^*−/−*^ mice were used. Each mouse was intravenously administered with SARS-CoV-2 E protein (0.5, 1, 2, or 4 μg per mouse, #RP01263, ABclonal Technology). For dose translation between laboratory animals and human in drug development, the dose in mouse were estimated by multiplying weight by 12.3^35^. By this criterion, when injected at 0.5 μg per mouse, the resulting concentration in the mice was comparable to 30 ng/ml of E protein in the serum of COVID-19 patients. PBS was used as a negative control. At 4 h post-administration, mice were anesthetized with 2% isoflurane, and a mixture of collagen (170 μg/kg) and epinephrine (60 μg/kg) in 100 μL PBS was injected into the tail vein to induce pulmonary embolism^34^. The mice were euthanized at 5 min post-injection, and the lungs were perfused and fixed in 4% formaldehyde solution for further hematoxylin/eosin and immunofluorescence staining.

### Inferior vena cava (IVC) stenosis model

An IVC stenosis model was performed as described^54^. Both WT mice and *CD36*^*−/−*^ mice were injected with PBS or SARS-CoV-2 E protein (4 μg per mouse, #RP01263, ABclonal Technology) through the tail vein for 4 h. After treatment, mice were anesthetized with 2% isoflurane, and then IVC and lateral branches were exposed after a careful laparotomy. After ligation of all lateral branches of IVC and separation of IVC from the aorta, a ligature with a 7-0 suture was fastened around the IVC over a blunted 30-gauge needle. The needle was then gently removed to restore partial blood flow and the abdominal cavity was closed completely. The mice were euthanized after 18 h, and the thrombus was harvested, photographed, and weighed. The thrombus was fixed in 4% formaldehyde for further immunofluorescence staining. To observe the effect of SB203580 (the p38 inhibitor) in the IVC model, mice were intraperitoneally pretreated with the SB203580 (10 mg/kg) or DMSO (negative control) 2 h before administration of the E protein.

### Immunofluorescence staining

Immunofluorescence assay of mouse lungs and thrombi from IVC was performed on 5 μm sections embedded in paraffin after fixation in 4% paraformaldehyde. The sections were subjected to de-paraffinization, heat-induced antigen retrieval and incubation with blocking solution as described^55^. For detection of CD41, rat anti-CD41 antibody (1:200, clone MWReg30, #553847, Becton Dickinson Biosciences) conjugated to Alexa Fluor 488 by using Alexa Fluor™ 488 Antibody Labeling Kit (#A20181, Invitrogen) was utilized. For detection of CD36 and E protein, mouse anti-CD36 (1 µg/mL, SMφ, #sc-7309, Santa Cruz) and rabbit anti-SARS-CoV-2 E antibodies (0.5 µg/mL, #NBP3-07060, Novus biologicals) were used, followed by incubation with secondary antibodies conjugated to CY3 (1:500, #AP132C, Sigma-Aldrich) and CY5 (1:1000, #AP500S, Sigma-Aldrich), respectively. Nuclear counterstaining was performed using DAPI (Invitrogen). The slides were examined by a Pannoramic MIDI scanner (3DHISTECH).

To perform platelet immunofluorescence staining, we isolated human platelets from both COVID-19 patients and healthy donors. These platelets were washed with PBS and cultured on glass slides coated with 20 μg/ml of fibrinogen. The cultures were maintained at 37 °C for 60 min. Subsequently, the spreading platelets were fixed, blocked, and stained using unconjugated anti-CD36 (1 µg/mL, FA6-152, #ab17044, Abcam) and anti-SARS-CoV-2 E (0.5 µg/mL, #NBP3-07060, Novus biologicals) antibodies. To visualize the staining, we utilized Alexa Fluor 488-conjugated (1:500, #A0423, Beyotime Biotechnology) and Alexa Fluor 594-conjugated secondary antibodies (1:200, #33212ES60, YEASEN), respectively. The resulting images were captured using a Zeiss microscope and analyzed using Image J software (v2.9.0, National Institutes of Health, Bethesda, MD).

### Statistical analysis

Statistical analyses were performed using SPSS 26 (SPSS, Chicago, IL). Data were presented as the means ± SEM or means ± SD as indicated. Data were tested for normality and equal variance before parametric analysis. The Student *t* test, 1-way ANOVA, 2-way ANOVA, Kruskal–Wallis test, Mann–Whitney *U* test, Spearman’s correlation test, Pearson’s correlation test or Fisher’s exact test were used, as appropriate. The statistical analysis method used for each experiment was indicated in the legend section. Graphs were generated using GraphPad Prism 9 Software. FlowJo V10.4.0 was used for flow cytometry analysis. All tests were 2-sided. *P* < 0.05 was considered statistically significant.

### Reporting summary

Further information on research design is available in the Nature Portfolio Reporting Summary linked to this article.

### Supplementary information

Supplementary Information

Peer Review File

Reporting Summary

### Source data

Source Data

## Data Availability

The RNA sequencing data generated in this study have been deposited in the GEO (Gene Expression Omnibus) database under accession code GSE214150. Clean reads were mapped to the reference genome (genome version # GCF_000001405.39_GRCh38;). The mass spectrometry proteomics data generated in this study have been deposited in the ProteomeXchange Consortium (http://proteomecentral.proteomexchange.org) via the iProX partner repository under accession code PXD037073. The data of the membrane proteins were searched against the UniprotKB Human Reference Proteome database (http://www.uniprot.org/, up to date as of December 10, 2021). All other data are included in the article and Supplemental materials. Source data are provided with this paper.
