# Supplementary material for: CD36 mediates SARS-CoV-2-envelope-protein-induced platelet activation and thrombosis
Source: Nat Commun. 2023 Aug 21;14:5077. doi: 10.1038/s41467-023-40824-7 (PMC10442425; doi:10.1038/s41467-023-40824-7)
Supplement: Supplementary file 1 — Supplementary Information [file 41467_2023_40824_MOESM1_ESM.pdf]

CD36 mediates SARS-CoV-2-Envelope-protein-induced platelet activation and thrombosis

Zihan Tang[1, *], Yanyan Xu[2, *], Yun Tan[3, *], Hui Shi[1, *], Peipei Jin[4, *], Yunqi Li[3, *], Jialin Teng[1], Honglei Liu[1], Haoyu Pan[1], Qiongyi Hu[1], Xiaobing Cheng[1], Junna Ye[1], Yutong Su[1], Yue Sun[1], Jianfen Meng[1], Zhuochao Zhou[1], Huihui Chi[1], Xuefeng Wang[4], Junling Liu[2], Yong Lu[5], Feng Liu[3, ‡], Jing Dai[4, ‡], Chengde Yang[1, ‡], Saijuan Chen[3, ‡], Tingting Liu[1, ‡]

[1]Department of Rheumatology and Immunology, Ruijin Hospital, Shanghai Jiao Tong University School of Medicine, No. 197 Ruijin Second Road, Shanghai, 200025, China.

[2]Department of Biochemistry and Molecular Cell Biology, Shanghai Jiao Tong University School of Medicine, 280 South Chongqing Road, Shanghai 200025, China.

[3]Shanghai Institute of Hematology, State Key Laboratory of Medical Genomics, National Research Center for Translational Medicine at Shanghai, Ruijin Hospital, Shanghai Jiao Tong University School of Medicine, Shanghai 200025, China.

[4]Department of Laboratory Medicine, Ruijin Hospital, Shanghai Jiao Tong University School of Medicine, Shanghai 200025, China.

[5]Department of Radiology, Ruijin Hospital, Shanghai Jiao Tong University School of Medicine, Shanghai, 200025 China

[‡]Corresponding authors: Tingting Liu (liutingting9905@163.com); Saijuan Chen (sjchen@stn.sh.cn); Chengde Yang (yangchengde@sina.com); Jing Dai (dj40572@rjh.com.cn); Feng Liu (fliu1978@126.com).

[*] These authors contributed equally.

Supplementary Figures

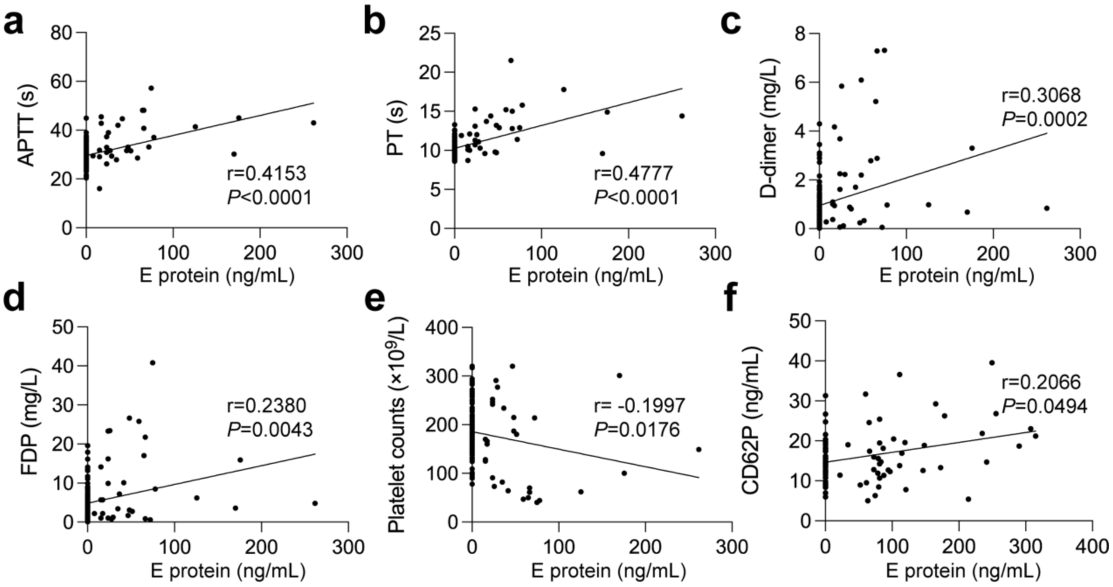

Supplementary Figure 1. Serum levels of the SARS-CoV-2 E protein correlate with abnormal coagulant parameters in COVID-19 patients. Two-tailed Spearman's or Pearson's rank correlation coefficient was used to measure the association between the level of the E protein and activated partial thromboplastin time (APTT) (**a**), prothrombin time (PT) (**b**), D-dimer (**c**), fibrinogen degradation products (FDP) (**d**), platelet counts (**e**), and CD62P (**f**) respectively. Source data are provided as a Source Data file.

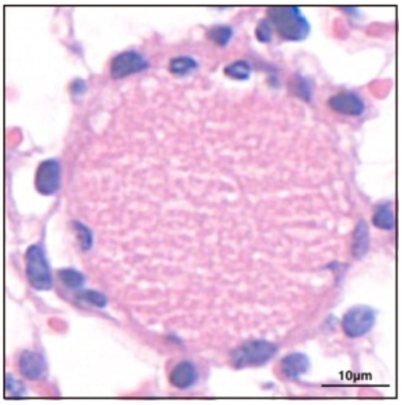

Supplementary Figure 2. Hematoxylin-eosin (HE) stained lung section of PE model. HE staining of thrombi in consecutive lung section, which showed the lung thrombi in the equivalent site in immunofluorescence staining of **Fig.2b**. Results were confirmed in five independent experiments. Scale bar=10μm.

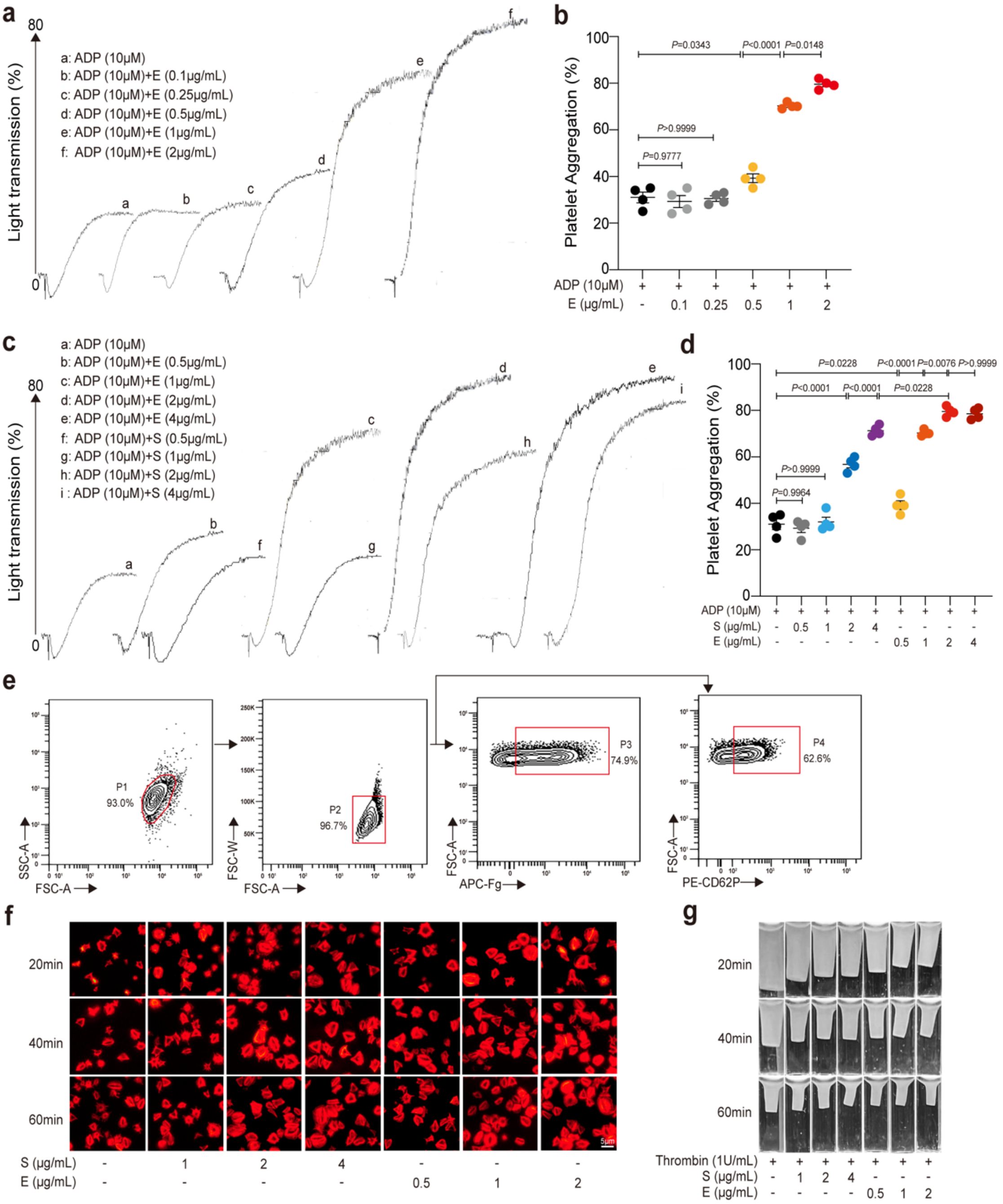

Supplementary Figure 3. The E protein activates platelets. a and **b** Aggregation of pretreated platelets with the E protein (0.1, 0.25, 0.5, 1, 2 μg/ml) was measured in response to ADP (10 μM) (n=4). **c** and **d** Aggregation of pretreated platelets with the E protein (0.5, 1, 2, 4 μg/ml) or the S protein (0.5, 1, 2, 4 μg/ml) was measured in response to ADP (10 μM) (n=4). **e** Platelet events in washed platelet samples were initially identified using polygonal gating on the FSC-A versus SSC-A scatter plot, excluding debris and aggregates by plotting FSC-A versus FSC-W. Subsequent analysis for P-selectin exposure and fibrinogen (Fg) binding were conducted based on the specific gate, respectively. **f** Pretreated platelets (0.5, 1, 2 μg/ml E or 1, 2, 4 μg/ml S) were

allowed to spread on immobilized Fg at 37°C for 20, 40, and 60 minutes. Representative field on platelet spreading. Scale bar=5μm. **g** Pretreated platelets were added into platelet-free plasma, and then clot retraction were induced by 1 U/ml thrombin. Photograph of clot retraction at 20, 40, and 60 minutes, respectively. Results in (**f** and **g**) were confirmed in four independent experiments. Data were analyzed by 1-way ANOVA with Tukey multiple-comparisons test (**b**, **d**). Data are presented as mean±SEM. Source data are provided as a Source Data file.

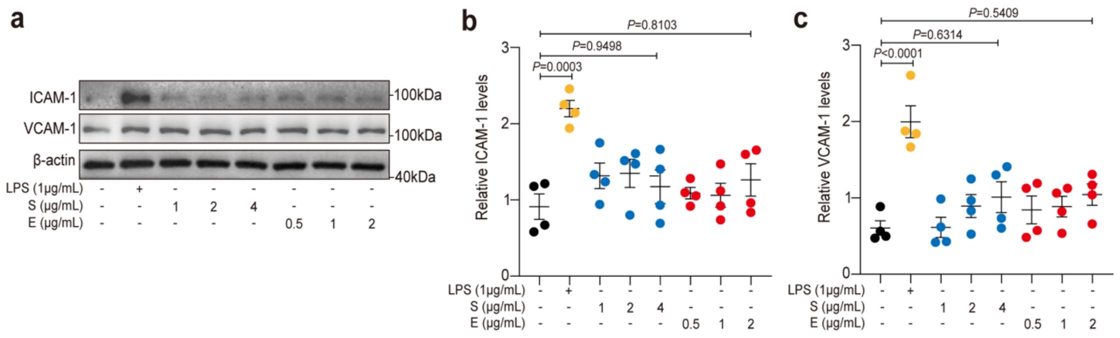

Supplementary Figure 4. The E protein did not activate endothelial cells. a-c The levels of intercellular adhesion molecule-1 (ICAM-1) and vascular cell adhesion molecule-1 (VCAM-1) in human umbilical vein endothelial cells (HUVECs) were detected by western blotting after incubated with the E protein (0.5, 1, 2 μg/ml), the S protein (1, 2, 4 μg/ml), or LPS (1 μg/ml) (n=4). Data were analyzed by 1-way ANOVA with Tukey multiple-comparisons test (**b**, **c**). The molecular weight markers are shown (**a**). Data are presented as mean±SEM. Source data are provided as a Source Data file.

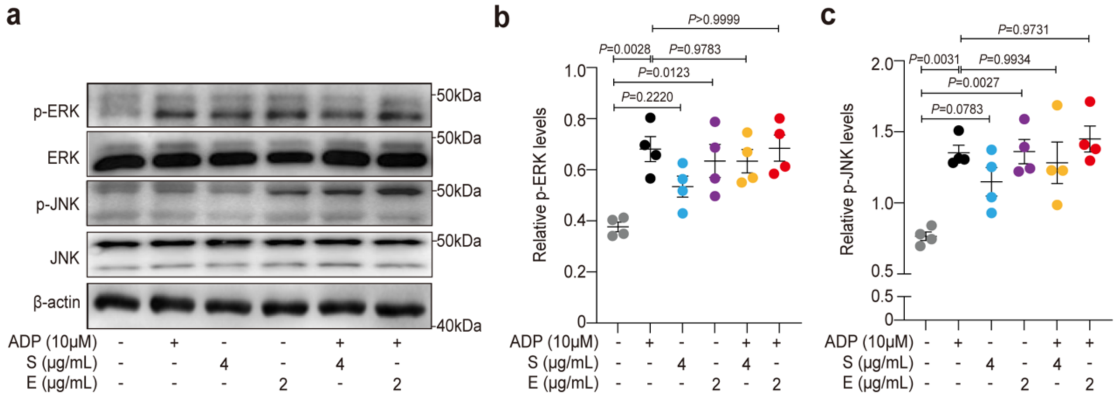

Supplementary Figure 5. The E protein did not enhance the phosphorylation of ERK or JNK in platelets. a-c The phosphorylated levels of ERK and JNK in platelets were detected by western blotting after incubated with the E protein (2 μg/ml) or the S protein (4 μg/ml) for 5 minutes at 37°C with or without ADP (10 μM) (n=4). The molecular weight markers are shown (**a**). Data were analyzed by 1-way ANOVA with Tukey multiple-comparisons test (**b**, **c**). Data are presented as mean±SEM. Source data are provided as a Source Data file.

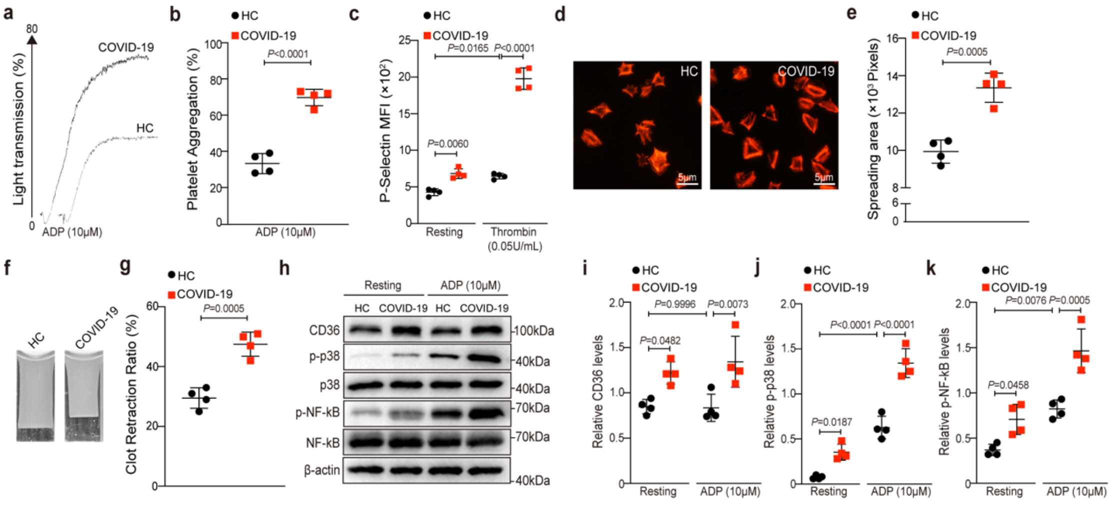

Supplementary Figure 6. Platelet activation is potentiated in COVID-19 patients. **a** and **b** Aggregation of washed platelets isolated from COVID-19 patients and healthy donors (HC) in response to ADP (10 μM) (n=4). **c** P-selectin exposure of platelets with or without stimulation of thrombin (0.05 U/mL) (n=4). **d** and **e** Spreading of platelets from COVID-19 patients and HC on immobilized fibrinogen (Fg) (n=4). Scale bar=5μm. **f** and **g** Clot retraction of platelets from COVID-19 patients and HC induced by 1 U/ml thrombin was photographed and analyzed at 30 minutes (n=4). **h-k** The relative levels of CD36 and phosphorylation levels of p38 and NF-*κ*B in human platelets isolated from COVID-19 patients and HC were detected by Western Blotting (n=4). The molecular weight markers are shown (**h**). Data were analyzed by unpaired 2-tailed Student's *t* test (**b**, **e**, **g**), or 2-way ANOVA with Tukey multiple-comparisons test (**c**, **i-k**). Data are presented as mean±SD. Source data are provided as a Source Data file.

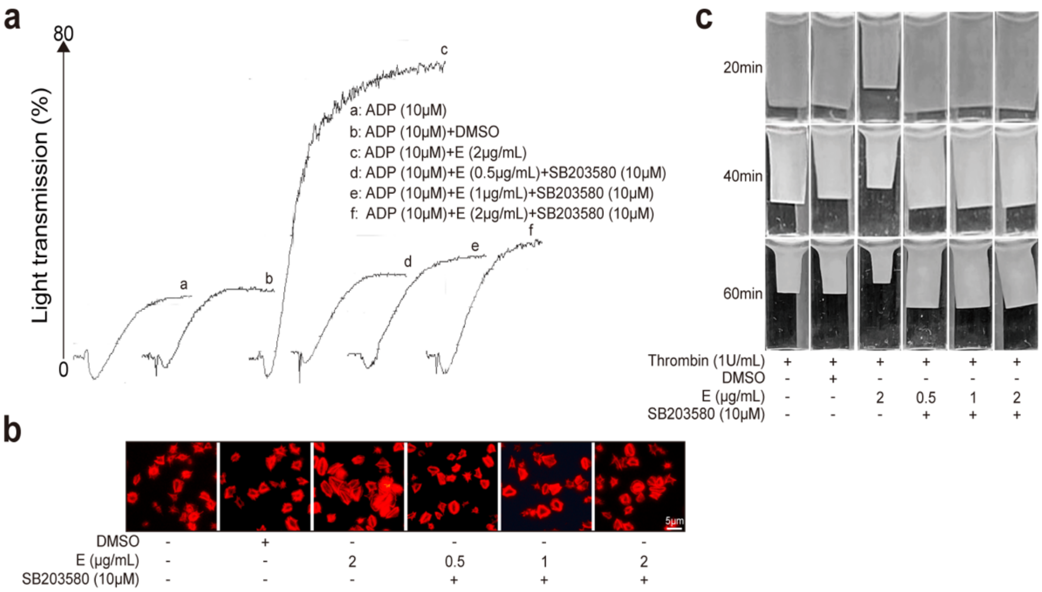

Supplementary Figure 7. SB203580 suppressed platelet activation induced by the E protein. a Potentiated platelet aggregation induced by the E protein combined with ADP 10μM was suppressed by SB203580. **b** Representative field on platelet spreading. Scale bar=5μm. **c** Photograph of clot retraction at 20, 40, and 60 minutes, respectively. Results in (**a-c**) were confirmed in four independent experiments.

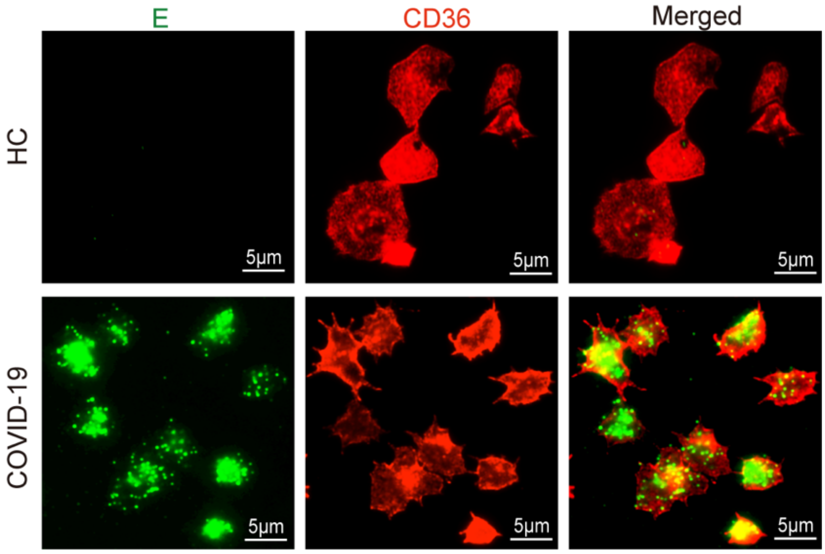

Supplementary Figure 8. The E protein and CD36 colocalized on platelets from COVID-19 patients. The colocalization of the E protein and CD36 on COVID-19 platelets. Immunofluorescence staining on platelets isolated from COVID-19 patients and HC was performed with anti-SARS-CoV-2 E protein (green), and anti-CD36 (red) antibodies. Scale bar=5μm. Results in were confirmed in three independent experiments.

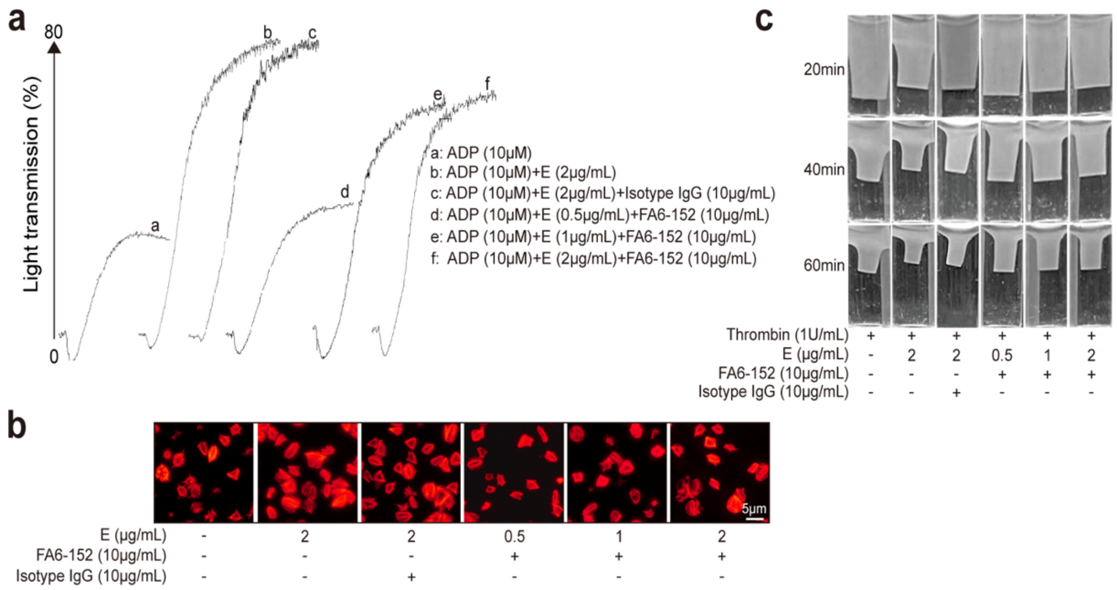

Supplementary Figure 9. Anti-CD36 antibody attenuated platelet activation enhanced by the E protein. a FA6-152 ameliorated enhanced platelet aggregation induced by the E protein. **b** Representative field on platelet spreading. Scale bar=5μm. **c** Photograph of clot retraction at 20, 40, and 60 minutes, respectively. Results in (**a-c**) were confirmed in four independent experiments.

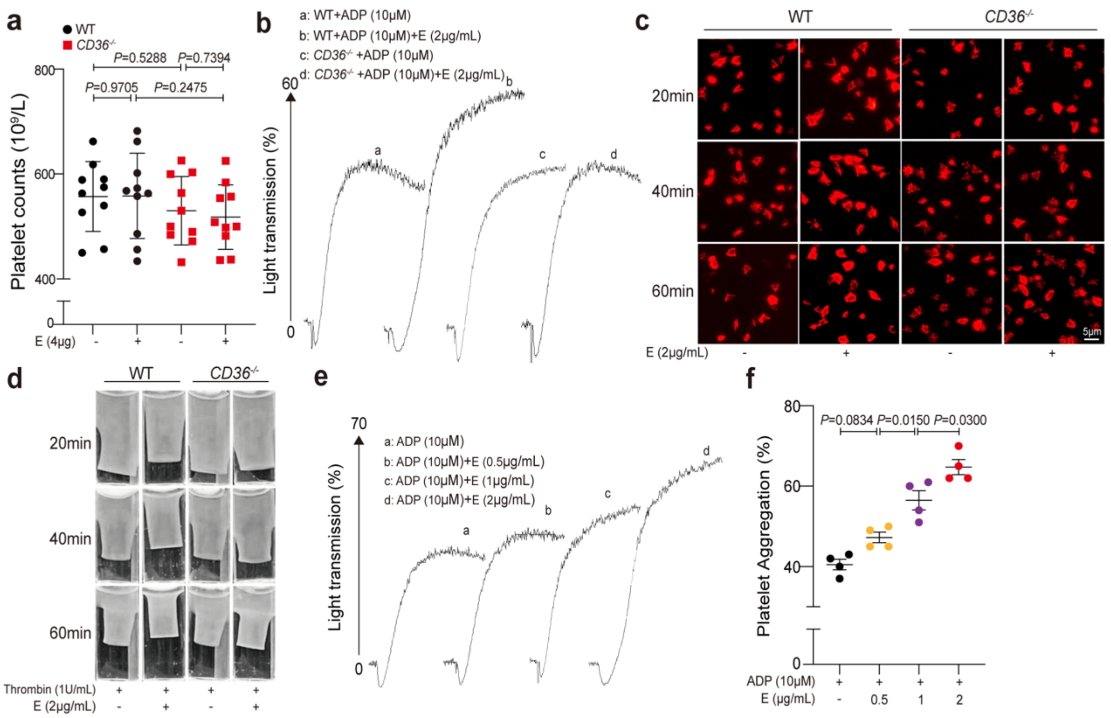

Supplementary Figure 10. *CD36* deficiency attenuates E protein-promoted platelet activation. a *CD36* deficiency and E protein did not influence the platelet counts. Platelet counts in WT mice and *CD36*$^{-/-}$ mice intravenously administrated with or without the E protein (4 μg per mouse) (n=10). Data are presented as mean±SD. **b** Aggregation enhanced by the E protein was attenuated in *CD36*$^{-/-}$ mice platelets in response to ADP. **c** The promoting effects of the E protein on platelet spreading were abolished in *CD36*$^{-/-}$ mouse platelets. Representative field on platelet spreading. Scale bar=5μm. **d** Potentiated clot retraction induced by the E protein was suppressed in *CD36*$^{-/-}$ mouse platelets. Photograph of clot retraction. Results in (**b-d**) were confirmed in four independent experiments. **e** and **f** Platelets from WT mice were incubated with the E protein (0.5, 1, 2μg/ml) or PBS for 5 minutes at 37°C, followed by stimulation with 10μM ADP (n=4). Data are presented as mean±SEM. Data were analyzed by 2-tailed Mann-Whitney *U* test (**a**) or 1-way ANOVA with Tukey multiple-comparisons test (**f**). Source data are provided as a Source Data file.

Supplementary Tables

Supplementary Table 1. Characteristics of COVID-19 patients and healthy donors.

	Healthy donors (n=51)	COVID-19 patients Non-severe (n=121)	COVID-19 patients Severe (n=24)	*P* value
Age (years)[#]	35 (34-46)	68 (57-77)	71 (66-83)	<0.001
Sex (F/M)	27/24	62/59	10/14	1.000
APTT[#]	/	29.1 (26.0-32.7)	33.0 (29.3-39.4)	0.004
D-Dimer[#]	/	0.6 (0.3-1.2)	1.0 (0.3-3.0)	0.121
FDP[#]	/	3.1 (1.7-6.4)	5.9 (2.3-14.7)	0.055
PT[#]	/	9.9 (9.5-10.8)	11.4 (10.5-12.8)	<0.001
Platelet counts[#]	/	183 (148-219)	154 (71-229)	0.036
Thrombotic events[‡]	/	5 (4.1)	7 (29.1)	0.001
Anti-platelet therapy				
Aspirin[‡]	/	12 (9.9)	0 (0)	/
Clopidogrel[‡]	/	13 (10.7)	3 (12.5)	/

[#] median (IQR); [‡] n (%); F, female; M, male. APTT, activated partial thromboplastin time (s); FDP, fibrin degradation products (mg/L); PT, prothrombin time (s); Platelet counts ($\times 10^9$/L). Data were analyzed by the Kruskal-Wallis test (age and sex), the Mann-Whitney *U* test (APTT, D-Dimer, FDP, PT and platelet counts), and Fisher's exact test (thrombotic events).

Supplementary Table 2. The information of membrane proteins analyzed by mass spectrometry.

Accession	Description	Coverage [%]	Peptides	PSMs	Unique Peptides	AAs	MW [kDa]	calc. pI	Abundances (Grouped): E1
P23229	Integrin alpha-6 OS=Homo sapiens OX=9606 GN=ITGA6 PE=1 SV=5	40	31	41	31	1130	126.5	6.61	920670936.3
P08514	Integrin alpha-IIb OS=Homo sapiens OX=9606 GN=ITGA2B PE=1 SV=3	32	26	36	26	1039	113.3	5.38	299092231.4
P12931	Proto-oncogene tyrosine-protein kinase Src OS=Homo sapiens OX=9606 GN=SRC PE=1 SV=3	58	24	40	19	536	59.8	7.42	2278943716
O14672	Disintegrin and metalloproteinase domain-containing protein 10 OS=Homo sapiens OX=9606 GN=ADAM10 PE=1 SV=1	37	22	50	22	748	84.1	7.77	1573995938
P05106	Integrin beta-3 OS=Homo sapiens OX=9606 GN=ITGB3 PE=1 SV=2	27	20	25	3	788	87	5.24	212612871.6
P48426	Phosphatidylinositol 5-phosphate 4-kinase type-2 alpha OS=Homo sapiens OX=9606 GN=PIP4K2A PE=1 SV=2	52	19	43	19	406	46.2	6.99	942511711.1
V5J3L2	STIM1 OS=Homo sapiens OX=9606 GN=STIM1 PE=2 SV=1	31	17	25	17	685	77.4	6.67	243915657.8
P40197	Platelet glycoprotein V OS=Homo sapiens OX=9606 GN=GP5 PE=1 SV=1	48	17	75	17	560	60.9	9.63	7196129031
B4DI38	Adenylyl cyclase-associated protein 1 OS=Homo sapiens OX=9606 PE=2 SV=1	43	17	24	17	452	49	8.21	174290270.2
P26038	Moesin OS=Homo sapiens OX=9606 GN=MSN PE=1 SV=3	23	16	21	9	577	67.8	6.4	144261185.4
Q14162	Scavenger receptor class F member 1 OS=Homo sapiens OX=9606 GN=SCARF1 PE=1 SV=3	27	14	14	14	830	87.3	6.37	86224514.69
A0A0C4DGZ8	Glycoprotein Ib (Platelet), alpha polypeptide OS=Homo sapiens OX=9606 GN=GP1BA PE=1 SV=1	25	14	20	14	626	68.9	6.68	815836684.1
Q8WWI5	Choline transporter-like protein 1 OS=Homo sapiens OX=9606 GN=SLC44A1 PE=1 SV=1	26	13	17	13	657	73.3	8.6	161114603.8
Q9UHQ9	NADH-cytochrome b5 reductase 1 OS=Homo sapiens OX=9606 GN=CYB5R1 PE=1 SV=1	56	13	20	13	305	34.1	9.38	288874514.3
Q5VY43	Platelet endothelial aggregation receptor 1 OS=Homo sapiens OX=9606 GN=PEAR1 PE=1 SV=1	17	11	15	11	1037	110.6	6.81	115553915.9

Accession	Description	Coverage [%]	Peptides	PSMs	Unique Peptides	AAs	MW [kDa]	calc. pI	Abundances (Grouped): E1
P05556	Integrin beta-1 OS=Homo sapiens OX=9606 GN=ITGB1 PE=1 SV=2	18	11	13	11	798	88.4	5.39	181037228
D9IX63	Glycoprotein IIIb OS=Homo sapiens OX=9606 GN=CD36 PE=2 SV=1	30	11	15	11	472	53	7.77	121726145.4
O00194	Ras-related protein Rab-27B OS=Homo sapiens OX=9606 GN=RAB27B PE=1 SV=4	65	11	18	9	218	24.6	5.52	440284162.6
Q9HCM2	Plexin-A4 OS=Homo sapiens OX=9606 GN=PLXNA4 PE=1 SV=4	6	9	9	9	1894	212.3	6.86	15241004.25
P10644	cAMP-dependent protein kinase type I-alpha regulatory subunit OS=Homo sapiens OX=9606 GN=PRKAR1A PE=1 SV=1	31	9	11	9	381	43	5.35	56358743.38
P98172	Ephrin-B1 OS=Homo sapiens OX=9606 GN=EFNB1 PE=1 SV=1	43	9	16	9	346	38	8.94	337861943
A0A024QZN4	Metavinculin OS=Homo sapiens OX=9606 GN=VCL PE=3 SV=1	10	8	9	8	1066	116.6	6.09	26642045.13
Q13443	Disintegrin and metalloproteinase domain-containing protein 9 OS=Homo sapiens OX=9606 GN=ADAM9 PE=1 SV=1	15	8	9	8	819	90.5	7.52	23964132.28
Q00013	55 kDa erythrocyte membrane protein OS=Homo sapiens OX=9606 GN=MPP1 PE=1 SV=2	26	8	10	8	466	52.3	7.37	64851052.13
P61224	Ras-related protein Rap-1b OS=Homo sapiens OX=9606 GN=RAP1B PE=1 SV=1	44	8	10	8	184	20.8	5.78	154968421.3
Q969P0	Immunoglobulin superfamily member 8 OS=Homo sapiens OX=9606 GN=IGSF8 PE=1 SV=1	15	7	8	7	613	65	8	37394371.63
Q9HCN6	Platelet glycoprotein VI OS=Homo sapiens OX=9606 GN=GP6 PE=1 SV=4	22	7	15	7	339	36.8	9.2	601223765.5
P21926	CD9 antigen OS=Homo sapiens OX=9606 GN=CD9 PE=1 SV=4	26	7	14	7	228	25.4	7.15	2382100735
P61006	Ras-related protein Rab-8A OS=Homo sapiens OX=9606 GN=RAB8A PE=1 SV=1	34	7	10	4	207	23.7	9.07	47431992.19
Q8IYS2	Uncharacterized protein KIAA2013 OS=Homo sapiens OX=9606 GN=KIAA2013 PE=1 SV=1	13	6	7	6	634	69.1	8.19	12203963.13

Accession	Description	Coverage [%]	Peptides	PSMs	Unique Peptides	AAs	MW [kDa]	calc. pI	Abundances (Grouped): E1
Q8N490	Probable hydrolase PNKD OS=Homo sapiens OX=9606 GN=PNKD PE=1 SV=2	23	6	8	6	385	42.8	9.09	17086938.38
Q86YW5	Trem-like transcript 1 protein OS=Homo sapiens OX=9606 GN=TREML1 PE=1 SV=2	41	6	11	6	311	32.7	6.05	249847579.6
Q9H3N1	Thioredoxin-related transmembrane protein 1 OS=Homo sapiens OX=9606 GN=TMX1 PE=1 SV=1	20	6	7	6	280	31.8	4.98	31097672.5
P54709	Sodium/potassium-transporting ATPase subunit beta-3 OS=Homo sapiens OX=9606 GN=ATP1B3 PE=1 SV=1	23	6	6	6	279	31.5	8.35	40830685.25
P42892	Endothelin-converting enzyme 1 OS=Homo sapiens OX=9606 GN=ECE1 PE=1 SV=2	9	5	5	5	770	87.1	5.88	15530356.88
Q9NV96	Cell cycle control protein 50A OS=Homo sapiens OX=9606 GN=TMEM30A PE=1 SV=1	16	5	5	5	361	40.7	8.59	18552544.88
Q9UIB8	SLAM family member 5 OS=Homo sapiens OX=9606 GN=CD84 PE=1 SV=1	20	5	6	5	345	38.8	7.06	46116334.63
Q15762	CD226 antigen OS=Homo sapiens OX=9606 GN=CD226 PE=1 SV=2	24	5	7	5	336	38.6	7.94	57686710
O95858	Tetraspanin-15 OS=Homo sapiens OX=9606 GN=TSPAN15 PE=1 SV=1	22	5	5	5	294	33.1	5.48	13967334.38
Q8NG11	Tetraspanin-14 OS=Homo sapiens OX=9606 GN=TSPAN14 PE=1 SV=1	23	5	7	5	270	30.7	6.84	95729585
Q96L08	Sushi domain-containing protein 3 OS=Homo sapiens OX=9606 GN=SUSD3 PE=1 SV=1	23	5	5	5	255	27.1	10.21	20049450.75
Q9P126	C-type lectin domain family 1 member B OS=Homo sapiens OX=9606 GN=CLEC1B PE=1 SV=2	21	5	6	5	229	26.6	8.5	257887168.1
P14770	Platelet glycoprotein IX OS=Homo sapiens OX=9606 GN=GP9 PE=1 SV=3	32	5	8	5	177	19	6.34	397444529.3
P01133	Pro-epidermal growth factor OS=Homo sapiens OX=9606 GN=EGF PE=1 SV=2	4	4	4	4	1207	133.9	5.85	34664545.75

Accession	Description	Coverage [%]	Peptides	PSMs	Unique Peptides	AAs	MW [kDa]	calc. pI	Abundances (Grouped): E1
P12318	Low affinity immunoglobulin gamma Fc region receptor II-a OS=Homo sapiens OX=9606 GN=FCGR2A PE=1 SV=4	21	4	5	4	317	35	6.68	38471335.13
Q86UF1	Tetraspanin-33 OS=Homo sapiens OX=9606 GN=TSPAN33 PE=1 SV=1	16	4	5	4	283	31.5	7.17	69194338.5
P29965	CD40 ligand OS=Homo sapiens OX=9606 GN=CD40LG PE=1 SV=1	21	4	4	4	261	29.3	8.31	16722897
Q92930	Ras-related protein Rab-8B OS=Homo sapiens OX=9606 GN=RAB8B PE=1 SV=2	25	4	7	1	207	23.6	9.07	1471318.125
P53801	Pituitary tumor-transforming gene 1 protein-interacting protein OS=Homo sapiens OX=9606 GN=PTTG1IP PE=1 SV=1	27	4	8	4	180	20.3	8.79	629763612
P30273	High affinity immunoglobulin epsilon receptor subunit gamma OS=Homo sapiens OX=9606 GN=FCER1G PE=1 SV=1	34	4	4	4	86	9.7	7.12	182104470.5
Q9Y2A7	Nck-associated protein 1 OS=Homo sapiens OX=9606 GN=NCKAP1 PE=1 SV=1	4	3	3	3	1128	128.7	6.62	10947293.13
Q8NDX1	PH and SEC7 domain-containing protein 4 OS=Homo sapiens OX=9606 GN=PSD4 PE=1 SV=2	2	3	3	3	1056	116.2	5.48	2293232.969
Q02413	Desmoglein-1 OS=Homo sapiens OX=9606 GN=DSG1 PE=1 SV=2	4	3	3	3	1049	113.7	5.03	4827916.25
O60331	Phosphatidylinositol 4-phosphate 5-kinase type-1 gamma OS=Homo sapiens OX=9606 GN=PIP5K1C PE=1 SV=2	6	3	3	3	668	73.2	5.29	3933281.625
Q6UX71	Plexin domain-containing protein 2 OS=Homo sapiens OX=9606 GN=PLXDC2 PE=1 SV=1	9	3	3	3	529	59.5	6.46	3795836.813
P06127	T-cell surface glycoprotein CD5 OS=Homo sapiens OX=9606 GN=CD5 PE=1 SV=2	7	3	4	3	495	54.5	8.21	4712301.75
O60884	DnaJ homolog subfamily A member 2 OS=Homo sapiens OX=9606 GN=DNAJA2 PE=1 SV=1	9	3	4	3	412	45.7	6.48	9544274.969
P31689	DnaJ homolog subfamily A member 1 OS=Homo sapiens OX=9606 GN=DNAJA1 PE=1 SV=2	9	3	4	3	397	44.8	7.08	7237897.125

Accession	Description	Coverage [%]	Peptides	PSMs	Unique Peptides	AAs	MW [kDa]	calc. pI	Abundances (Grouped): E1
Q96S97	Myeloid-associated differentiation marker OS=Homo sapiens OX=9606 GN=MYADM PE=1 SV=2	16	3	3	3	322	35.3	8.15	9337165.875
O75954	Tetraspanin-9 OS=Homo sapiens OX=9606 GN=TSPAN9 PE=1 SV=1	15	3	9	3	239	26.8	7.68	130426899.4
Q6ZUX7	LHFPL tetraspan subfamily member 2 protein OS=Homo sapiens OX=9606 GN=LHFPL2 PE=1 SV=2	19	3	3	3	228	24.5	6.49	19366436.5
P51148	Ras-related protein Rab-5C OS=Homo sapiens OX=9606 GN=RAB5C PE=1 SV=2	18	3	3	3	216	23.5	8.41	11121116.13
P11234	Ras-related protein Ral-B OS=Homo sapiens OX=9606 GN=RALB PE=1 SV=1	19	3	3	3	206	23.4	6.62	5900523.25
Q9NYL4	Peptidyl-prolyl cis-trans isomerase FKBP11 OS=Homo sapiens OX=9606 GN=FKBP11 PE=1 SV=1	22	3	3	3	201	22.2	9.39	8403935
P13224	Platelet glycoprotein Ib beta chain OS=Homo sapiens OX=9606 GN=GP1BB PE=1 SV=1	15	3	6	3	206	21.7	9.31	839387472
Q12974	Protein tyrosine phosphatase type IVA 2 OS=Homo sapiens OX=9606 GN=PTP4A2 PE=1 SV=1	28	3	3	3	167	19.1	8.37	7594738.125
Q9UHN6	Cell surface hyaluronidase OS=Homo sapiens OX=9606 GN=CEMIP2 PE=1 SV=1	2	2	2	2	1383	154.3	8.15	3436520
Q8IY33	MICAL-like protein 2 OS=Homo sapiens OX=9606 GN=MICALL2 PE=1 SV=1	2	2	2	2	904	97.4	9.57	3912160.563
Q9ULF5	Zinc transporter ZIP10 OS=Homo sapiens OX=9606 GN=SLC39A10 PE=1 SV=2	2	2	2	2	831	94.1	6.76	2406902
P50148	Guanine nucleotide-binding protein G(q) subunit alpha OS=Homo sapiens OX=9606 GN=GNAQ PE=1 SV=4	9	2	3	2	359	42.1	5.68	2491893.063
Q9H813	Proton-activated chloride channel OS=Homo sapiens OX=9606 GN=PACC1 PE=1 SV=1	8	2	3	2	350	40	8.88	3855691.875
Q9H0U3	Magnesium transporter protein 1 OS=Homo sapiens OX=9606 GN=MAGT1 PE=1 SV=1	6	2	2	2	335	38	9.63	3908422

Accession	Description	Coverage [%]	Peptides	PSMs	Unique Peptides	AAs	MW [kDa]	calc. pI	Abundances (Grouped): E1
P21731	Thromboxane A2 receptor OS=Homo sapiens OX=9606 GN=TBXA2R PE=1 SV=3	5	2	2	2	343	37.4	9.91	13681019.5
Q96QS1	Tetraspanin-32 OS=Homo sapiens OX=9606 GN=TSPAN32 PE=2 SV=1	8	2	2	2	320	34.6	8.4	8148241
Q6PI78	Transmembrane protein 65 OS=Homo sapiens OX=9606 GN=TMEM65 PE=1 SV=2	9	2	2	2	240	25.5	8.6	4830432
P62491	Ras-related protein Rab-11A OS=Homo sapiens OX=9606 GN=RAB11A PE=1 SV=3	10	2	2	2	216	24.4	6.57	8707227.25
P60953	Cell division control protein 42 homolog OS=Homo sapiens OX=9606 GN=CDC42 PE=1 SV=2	14	2	2	2	191	21.2	6.55	1772541.875
