# Supplementary material for: CD36 mediates SARS-CoV-2-envelope-protein-induced platelet activation and thrombosis
Source: Nat Commun. 2023 Aug 21;14:5077. doi: 10.1038/s41467-023-40824-7 (PMC10442425; doi:10.1038/s41467-023-40824-7)
Supplement: Supplementary file 3 — Reporting Summary [file 41467_2023_40824_MOESM3_ESM.pdf]

nature portfolio

Corresponding author(s): Tingting Liu, Saijuan Chen, Chengde Yang, Jing Dai, Feng Liu

Last updated by author(s): Jul 30, 2023

Reporting Summary

Nature Portfolio wishes to improve the reproducibility of the work that we publish. This form provides structure for consistency and transparency in reporting. For further information on Nature Portfolio policies, see our Editorial Policies and the Editorial Policy Checklist.

Statistics

For all statistical analyses, confirm that the following items are present in the figure legend, table legend, main text, or Methods section.

n/a	Confirmed	
☐	☒	The exact sample size (*n*) for each experimental group/condition, given as a discrete number and unit of measurement
☐	☒	A statement on whether measurements were taken from distinct samples or whether the same sample was measured repeatedly
☐	☒	The statistical test(s) used AND whether they are one- or two-sided<br>*Only common tests should be described solely by name; describe more complex techniques in the Methods section.*
☒	☐	A description of all covariates tested
☐	☒	A description of any assumptions or corrections, such as tests of normality and adjustment for multiple comparisons
☐	☒	A full description of the statistical parameters including central tendency (e.g. means) or other basic estimates (e.g. regression coefficient) AND variation (e.g. standard deviation) or associated estimates of uncertainty (e.g. confidence intervals)
☐	☒	For null hypothesis testing, the test statistic (e.g. *F*, *t*, *r*) with confidence intervals, effect sizes, degrees of freedom and *P* value noted<br>*Give P values as exact values whenever suitable.*
☒	☐	For Bayesian analysis, information on the choice of priors and Markov chain Monte Carlo settings
☒	☐	For hierarchical and complex designs, identification of the appropriate level for tests and full reporting of outcomes
☐	☒	Estimates of effect sizes (e.g. Cohen's *d*, Pearson's *r*), indicating how they were calculated

Our web collection on statistics for biologists contains articles on many of the points above.

Software and code

Policy information about availability of computer code

Data collection	RNA sequencing was performed on the BGISEQ500 platform (BGI-Shenzhen, China) and the sequencing reads were filtered with SOAPnuke (v1.5.2). Clean reads were mapped using HISAT2 (v2.0.4).<br>The LC-MS/MS spectra were searched using MASCOT engine (Matrix Science, London, UK; version 2.2).<br>SPR data was obtained by Biacore 8K (GE Healthcare) Instrument.<br>For data collection of flow cytometry, BD FACSDiva software (version 8.0.1) was used. For ELISA, data were collected by Gen5 CHS software (version 2.09). For immunoblotting, images were collected by GeneSys software (v1.8.6.0). Platelet aggregation was recorded by using Aggrolink software (ChronoLog, USA). Image acquisition of platelet spreading and platelet immunofluorescence staining were done using a Zeiss microscope. Immunofluorescence images of mouse lung and thrombi from inferior vena cava (IVC) were collectec by a Pannoramic MIDI scanner (3DHISTECH).
Data analysis	Differential expression genes (DEGs) analysis for RNA sequencing was carried out by DESeq2 (v1.4.5).<br>Reference-substrated curves were fitted to a 1:1 binding model using Biacore Insight Evaluation Software (v3.0.12).<br>Statistical analyses were performed using SPSS 26 (SPSS, Chicago, IL). Graphs were generated using GraphPad Prism 9 Software. FlowJo V10.4.0 was used for flow cytometry analysis. Data of western blot and platelet spreading were analyzed using ImageJ (v2.9.0).

For manuscripts utilizing custom algorithms or software that are central to the research but not yet described in published literature, software must be made available to editors and reviewers. We strongly encourage code deposition in a community repository (e.g. GitHub). See the Nature Portfolio guidelines for submitting code & software for further information.

Data

Policy information about availability of data

All manuscripts must include a data availability statement. This statement should provide the following information, where applicable:

- Accession codes, unique identifiers, or web links for publicly available datasets
- A description of any restrictions on data availability
- For clinical datasets or third party data, please ensure that the statement adheres to our policy

RNA-seq data are deposited in the GEO database under the accession code GSE214150. Clean reads were mapped to the reference genome (genome version # GCF_000001405.39_GRCh38.p13). The mass spectrometry proteomics data are deposited to the ProteomeXchange Consortium (http://proteomecentral.proteomexchange.org) via the iProX partner repository with the dataset identifier PXD037073. The data of the membrane proteins were searched against the UniprotKB Human Reference Proteome database (http://www.uniprot.org/, up to date as of December 10, 2021). All other data are included in the article and Supplemental materials. Source data are provided with this paper.

Research involving human participants, their data, or biological material

Policy information about studies with human participants or human data. See also policy information about sex, gender (identity/presentation), and sexual orientation and race, ethnicity and racism.

Reporting on sex and gender	236 COVID-19 patients: 125 males and 111 females; 51 healthy donors: 24 males and 27 females. We did not perform sex- and gender-based analyses. In this study, sex was not included as a variable in the study design and analysis due to the absence of conclusive evidence suggesting a sex bias in COVID-19 infection at the time of the study. As a result, the researchers chose a consecutive enrollment approach and did not conduct sex selection when enrolling both COVID-19 patients and healthy controls. Since sex was not a targeted variable in the study, the data related to sex-specific differences were not collected or analyzed separately.
Reporting on race, ethnicity, or other socially relevant groupings	All the participants were Asians.
Population characteristics	145 COVID-19 patients and 51 healthy donors were enrolled. All COVID-19 patients met the diagnostic criteria for COVID-19, and SARS-CoV-2 infection was confirmed by reverse transcription polymerase chain reaction (RT-PCR). COVID-19 symptoms were diagnosed as mild (common flu-like symptoms without pneumonia), moderate (mild pneumonia), severe (respiratory frequency ≥30/min, blood oxygen saturation <93% at rest, the ratio of arterial oxygen partial pressure to fractional inspired oxygen [PaO2/FiO2] <300, and/or pulmonary inflammation progressing >50% within 24 to 48 hours), or critical (respiratory failure, septic shock, and/or multiple organ dysfunction)[Bmj 368, m1091 (2020)]. Subsequently, patients with mild or moderate symptoms were classified into non-severe group, and those with severe or critical symptoms into severe group. 91 COVID-19 patients who had not undergone antiplatelet drug therapy in the one month prior to enrollment were recruited.
Recruitment	All participants meeting the criteria were recruited consecutively with no any bias. All of them gave written informed consent for the provision of specimens for testing, further diagnosis, and scientific research. Blood samples from the COVID-19 patients at the time of hospital admission or healthy donors were collected. After centrifugation, serum or plasma was separated and kept frozen until use. For platelet-related experiments, human washed platelet were separated from platelet-rich plasma (PRP) by centrifugation at 1,000g for 10 minutes and resuspended in Tyrode buffer.
Ethics oversight	All participants were recruited under study protocols approved by the Institutional Review Board of Ruijin Hospital (ID: 2022-71), Shanghai Jiao Tong University School of Medicine. Written informed consent was obtained from all participants.

Note that full information on the approval of the study protocol must also be provided in the manuscript.

Field-specific reporting

Please select the one below that is the best fit for your research. If you are not sure, read the appropriate sections before making your selection.

☒ Life sciences ☐ Behavioural & social sciences ☐ Ecological, evolutionary & environmental sciences

For a reference copy of the document with all sections, see nature.com/documents/nr-reporting-summary-flat.pdf

Life sciences study design

All studies must disclose on these points even when the disclosure is negative.

Sample size	For human platelet function assays and measurement of E protein and CD62P, preliminary experiments were performed to determine proper sample size. 145 COVID-19 patients and 51 healthy controls were enrolled for the measurement of serum E protein. Among them, 4 COVID-19 patients and 4 healthy controls were recruited for human platelet function assay, and 3 healthy donors were included for platelet RNA-sequencing. 91 COVID-19 patients were recruited for measurement of E protein and CD62P.<br>Based on the previous studies the expected variance would allow significant results with a 10% effect size, alpha=0.05, beta=0.2 at group size of at least 2-3 mice. Data were analyzed when a predetermined power to detect a 10% points difference was reached. The specific number of

mice utilized in each experiment is indicated in the respective figure legends.

Data exclusions	Measured values were excluded in cases of technical failure during the experiment.
Replication	Experimental points 'n'-values as biological replication are indicated in each Figure legend. The entire experiments were repeated at least three times.
Randomization	A total of 236 COVID-19 patients and 51 healthy donors were consecutively included in the present study. There is no self-selection or group allocation of patient samples for correlation analysis.<br>All animal experiments were performed in a randomized manner.
Blinding	The investigators were blinded to group allocations during data collection and/or analysis. All the experiments were performed in a blinded manner. Representative pathology images were selected that represent the mean score after blinded quantification.

Reporting for specific materials, systems and methods

We require information from authors about some types of materials, experimental systems and methods used in many studies. Here, indicate whether each material, system or method listed is relevant to your study. If you are not sure if a list item applies to your research, read the appropriate section before selecting a response.

Materials & experimental systems

n/a	Involved in the study	
☐	☒	Antibodies
☒	☐	Eukaryotic cell lines
☒	☐	Palaeontology and archaeology
☐	☒	Animals and other organisms
☒	☐	Clinical data
☒	☐	Dual use research of concern
☒	☐	Plants

Methods

n/a	Involved in the study	
☒	☐	ChIP-seq
☐	☒	Flow cytometry
☒	☐	MRI-based neuroimaging

Antibodies

Antibodies used

Phospho-p38 MAPK Rabbit mAb (clone D3F9, #4511, Cell Signaling Technology)
p38 MAPK Rabbit mAb (clone D13E1, #8690, Cell Signaling Technology)
Phospho-NF-κB p65 (Ser536) Rabbit mAb (clone 93H1, #3033, Cell Signaling Technology)
NF-κB p65 Rabbit mAb (clone D14E12, #8242, Cell Signaling Technology)
Phospho-Erk1/2 Rabbit mAb (clone D13.14.4E, #4370, Cell Signaling Technology)
Erk1/2 Rabbit mAb (clone 137F5, #4695, Cell Signaling Technology)
Phospho-JNK Rabbit mAb (clone 81E11, #4668, Cell Signaling Technology)
SAPK/JNK Rabbit mAb (clone 56G8, #9258, Cell Signaling Technology)
β-Actin Rabbit Antibody (#AF5003, Beyotime Biotechnology)
Human CD36/SR-B3 Antibody (Clone #255619, #MAB1955, R&D Systems)
CD54/ICAM-1 Antibody (#4915,Cell Signaling Technology)
VCAM-1 Rabbit mAb (clone E1E8X, #13662, Cell Signaling Technology)
Anti-CD36 mouse mAb (FA6-152, #ab17044, Abcam)
Anti-CD36 mouse mAb (SMϕ, #sc-7309, Santa Cruz Biotechnology)
Goat Anti-Human IgG Fc (HRP)(#ab97225, Abcam)
Mouse anti His-Tag mAb (clone AMC0149, #AE003, ABclonal Technology)
Mouse mAb IgG1 Isotype Control (clone G3A1, #5415, Cell Signaling Technology)
Rabbit IgG isotype control (clone ARC5105-03, #AC042, ABclonal Technology)
SARS-CoV-2 Envelope Antibody (#NBP3-07959, Novus biologicals)
SARS-CoV-2 Envelope Antibody (#NBP3-07060, Novus biologicals)
Biotin Rabbit polyclonal to SARS-CoV-2 envelope protein (#ab284658, Abcam)
HRP-conjugated anti-rabbit IgG (#7074S, Cell Signaling Technology)
Anti-rat IgG, HRP-linked Antibody (#7077, Cell Signaling Technology)
HRP-labeled Goat Anti-Mouse IgG(H+L)(#A0216, Beyotime Biotechnology)
PE Mouse Anti-Human CD62P (clone AK-4, #555524, Becton Dickinson Biosciences)
Rat Anti-Mouse CD41 (clone MWReg30, #553847, Becton Dickinson Biosciences)
APC Mouse Anti-Human CD41a (clone HIP8, #559777, Becton Dickinson Biosciences)
Alexa Fluor 488-labeled Goat Anti-Rabbit IgG (H+L)(#A0423, Beyotime Biotechnology)
Alexa Fluor 594 AffiniPure Goat Anti-Mouse IgG (H+L) (#33212ES60, YEASEN)
Goat Anti-Mouse Antibody, Cy5 conjugate (#AP500S, Sigma-Aldrich)
Goat Anti-Rabbit Antibody, Cy3 conjugate (#AP132C, Sigma-Aldrich)

Validation

Western Blotting:
Phospho-p38 MAPK Rabbit mAb (1:1000, clone D3F9, #4511, Cell Signaling Technology); Species reactivity: human, mouse; Tested applications: WB, IHC, IF, Flow cytometry.
p38 MAPK Rabbit mAb (1:1000, clone D13E1, #8690, Cell Signaling Technology); Species reactivity: human, mouse; Tested applications: WB, IHC, IF, Flow cytometry.

Phospho-NF-κB p65 (Ser536) Rabbit mAb (1:1000, clone 93H1, #3033, Cell Signaling Technology); Species reactivity: human, mouse; Tested applications: WB, IP, IF, Flow cytometry.
NF-κB p65 Rabbit mAb (1:1000, clone D14E12, #8242, Cell Signaling Technology); Species reactivity: human, mouse; Tested applications: WB, IP, IF, Flow cytometry.
Phospho-Erk1/2 Rabbit mAb (1:1000, clone D13.14.4E, #4370, Cell Signaling Technology); Species reactivity: human, mouse; Tested applications: WB, IHC, IF, IP, Flow cytometry.
Erk1/2 Rabbit mAb (1:1000, clone 137F5, #4695, Cell Signaling Technology); Species reactivity: human, mouse; Tested applications: WB, IHC, IF, IP, Flow cytometry.
Phospho-JNK Rabbit mAb (1:1000, clone 81E11, #4668, Cell Signaling Technology); Species reactivity: human, mouse; Tested applications: WB, IHC, IP.
SAPK/JNK Rabbit mAb (1:1000, clone 56G8, #9258, Cell Signaling Technology); Species reactivity: human, mouse; Tested applications: WB.
β-Actin Rabbit Antibody (1:2000, #AF5003, Beyotime Biotechnology); Species reactivity: human, mouse; Tested applications: WB, IF, IHC.
CD54/ICAM-1 Antibody (1:1000, #4915, Cell Signaling Technology); Species reactivity: human; Tested applications: WB.
VCAM-1 Rabbit mAb (1:1000, clone E1E8X, #13662, Cell Signaling Technology); Species reactivity: human; Tested applications: WB, IP, Flow cytometry.
Human CD36/SR-B3 Antibody (1 μg/mL, #MAB1955, R&D Systems); Species reactivity: human; Tested applications: WB.
Anti-CD36 mouse mAb (1 μg/mL, SMϕ, #sc-7309, Santa Cruz Biotechnology); Species reactivity: human, mouse, rat; Tested applications: WB, IP, IF, IHC (P), Flow cytometry.
Mouse anti His-Tag mAb (1:2000, clone AMC0149, #AE003, ABclonal Technology); Species independent; Tested applications: WB, IP.
Goat Anti-Human IgG Fc (HRP)(1:3000, #ab97225, Abcam); Species reactivity: human; Tested applications: WB, ICC, IHC (P), ELISA.
Biotin Rabbit polyclonal to SARS-CoV-2 envelope protein (1 μg/mL, #ab284658, Abcam); Species reactivity: SARS-CoV-2; Tested applications: ELISA.
HRP-conjugated anti-rabbit IgG (1:5000, #7074S, Cell Signaling Technology); Species reactivity: rabbit; Tested applications: WB.
HRP-labeled Goat Anti-Mouse IgG(H+L)(1:1000, #A0216, Beyotime Biotechnology); Species reactivity: mouse; Tested applications: WB, ELISA, IHC.
Anti-rat IgG, HRP-linked Antibody (1:3000, #7077, Cell Signaling Technology); Species reactivity: rat; Tested applications: WB.

Co-immunoprecipitation:
Mouse mAb IgG1 Isotype Control (10 μg/mL, clone G3A1, #5415, Cell Signaling Technology); Species reactivity: N/A; Tested applications: IP, IHC, IF, Flow cytometry.
Anti-CD36 mouse mAb (10 μg/mL, FA6-152, #ab17044, Abcam); Species reactivity: human; Tested applications: IHC-P, IHC-Fr, Flow cytometry. This antibody has been validated in IP experiments (Circulation, 143(1), 45–61.).

In vitro treatment:
Mouse mAb IgG1 Isotype Control (10 μg/mL, clone G3A1, #5415, Cell Signaling Technology); (10 μg/mL, clone G3A1, #5415, Cell Signaling Technology); Species reactivity: N/A; Tested applications: IP, IHC, IF, Flow cytometry.
Anti-CD36 mouse mAb (10 μg/mL, FA6-152, #ab17044, Abcam); Species reactivity: human; Tested applications: IHC-P, IHC-Fr, Flow cytometry. This antibody has been validated in in vitro experiments (Circulation, 143(1), 45–61.).

ELISA:
SARS-CoV-2 Envelope Antibody (4 μg/mL,#NBP3-07959, Novus biologicals); Species reactivity: SARS-CoV-2; Tested applications: WB, ELISA, IHC.
HRP-conjugated anti-rabbit IgG (1:1000, #7074S, Cell Signaling Technology); Species reactivity: rabbit; Tested applications: WB.
Human P-Selectin/CD62P DuoSet ELISA (#DY137, R&D Systems); Species reactivity: human; Tested applications: ELISA.

Flow cytometry:
PE Mouse Anti-Human CD62P (1:100, clone AK-4, #555524, Becton Dickinson Biosciences); Species reactivity: human; Tested applications: Flow cytometry.
Fibrinogen from human plasma, Alexa Fluor® 647 conjugate (1:50, #F35200, Life Technologies); Species reactivity: human; Tested applications: Flow cytometry.
APC Mouse Anti-Human CD41a (1:100, clone HIP8, #559777, Becton Dickinson Biosciences); Species reactivity: human; Tested applications: Flow cytometry.
FITC Mouse Anti-Human CD45 (1:100, clone HI30(RUO), #555482, Becton Dickinson Biosciences); Species reactivity: human; Tested applications: Flow cytometry.

Immunofluorescence staining:
Rat Anti-Mouse CD41 (1:200, clone MWReg30, #553847, Becton Dickinson Biosciences); Species reactivity: mouse; Tested applications: IHC-F, IP, Depletion, Flow cytometry.
Alexa Fluor™ 488 Antibody Labeling Kit (100 μg antibody/1 reaction, #A20181, Invitrogen); Tested applications: antibody labeling.
SARS-CoV-2 Envelope Antibody (0.5 μg/mL, #NBP3-07060, Novus biologicals); Species reactivity: SARS-CoV-2; Tested applications: WB, ELISA, IHC.
Rabbit IgG isotype control (0.5 μg/mL, clone ARC5105-03, #AC042, ABclonal Technology); Species reactivity: N/A; Tested applications: IHC-P, IF, IP.
Anti-CD36 mouse mAb (1 μg/mL, SMϕ, #sc-7309, Santa Cruz Biotechnology); Species reactivity: human, mouse, rat; Tested applications: WB, IP, IF, IHC (P), Flow cytometry.
Anti-CD36 mouse mAb (1 μg/mL, FA6-152, #ab17044, Abcam); Species reactivity: human; Tested applications: IHC-P, IHC-Fr, Flow cytometry.
Mouse mAb IgG1 Isotype Control (1 μg/mL, clone G3A1, #5415, Cell Signaling Technology); Species reactivity: N/A; Tested applications: IP, IHC, IF, Flow cytometry.
Alexa Fluor 488-labeled Goat Anti-Rabbit IgG (H+L)(1:500, #A0423, Beyotime Biotechnology); Species reactivity: rabbit; Tested applications: IF.
Alexa Fluor 594 AffiniPure Goat Anti-Mouse IgG (H+L) (1:200, #33212ES60, YEASEN); Species reactivity: mouse; Tested applications: IF.

Goat Anti-Mouse Antibody, Cy5 conjugate (1:1000, #AP500S, Sigma-Aldrich); Species reactivity: mouse; Tested applications: IF.
Goat Anti-Rabbit Antibody, Cy3 conjugate (1:500, #AP132C, Sigma-Aldrich); Species reactivity: rabbit; Tested applications: IF.

Animals and other research organisms

Policy information about studies involving animals; ARRIVE guidelines recommended for reporting animal research, and Sex and Gender in Research

Laboratory animals	CD36 knockout (CD36-/-) on C57BL/6 genetic background and C57BL/6 control mice (both male and female, 6-8 weeks, 20-25g) were purchased from Cyagen Bioscience, Inc.. Mice were housed in a specific pathogen-free environment on 12-hour light/dark cycles with free access to food and water, ambient temperature 22-24?, and humidity 50-70%.
Wild animals	No wild animals were used in the study.
Reporting on sex	Both female and male mice were used in this study. Sex-matched CD36 knockout (CD36-/-) on C57BL/6 genetic background and C57BL/6 control mice were used in platelet function assay, pulmonary embolism model, inferior vena cava (IVC) stenosis model and tail bleeding assay. In this study involving a mouse experiment, the consideration of sex as a variable in the study design and analysis was omitted due to the limited availability of evidence suggesting sex-based differences in COVID-19 pathogenesis in mice. As a result, we presented the data in an aggregated manner for sex.
Field-collected samples	The study did not involve samples collected from the field.
Ethics oversight	All animal procedures were performed in accordance and approved by Shanghai Jiao Tong University School of Medicine. In this study, all animal handling, welfare, monitoring, and euthanasia practices were performed in strict accordance with the ethical guidelines. More specifically, mice were housed in a specific pathogen-free environment on 12-hour light/dark cycles with free access to food and water, ambient temperature 22-24?, and humidity 50-70%. Mice were handled using tunnels or an open hand approach instead of being picked up by the tail, aiming to minimize stress and promote their well-being. Animals were regularly monitored by qualified personnel to ensure their health and well-being. If necessary, euthanasia was performed using CO2 inhalation, following approved protocols.

Note that full information on the approval of the study protocol must also be provided in the manuscript.

Flow Cytometry

Plots

Confirm that:

☒ The axis labels state the marker and fluorochrome used (e.g. CD4-FITC).

☒ The axis scales are clearly visible. Include numbers along axes only for bottom left plot of group (a 'group' is an analysis of identical markers).

☒ All plots are contour plots with outliers or pseudocolor plots.

☒ A numerical value for number of cells or percentage (with statistics) is provided.

Methodology

Sample preparation	Human blood was collected from the antecubital vein into a blood collection tube containing buffered sodium citrate. Washed platelets were separated from platelet-rich plasma (PRP) by centrifugation at 1000g for 10 minutes and resuspended in Tyrode buffer [Blood. 2006 Oct 15;108(8):2596-603; Blood. 2010 Oct 7;116(14):2579-81.].Washed human platelets were incubated with APC-conjugated anti-CD41 antibodies and FITC-conjugated anti-CD45 antibodies for 20 min at room temperature to determine the purity. For P-selection exposure and fibrinogen (Fg) binding assay, pretreated platelets from COVID-19 patients and healthy controls (HC) were incubated with PE-conjugated anti-CD62P antibodies and AF647 conjugated Fg for 20 min at room temperature with or without thrombin and measured by a flow cytometer. To detect the effect of S or E protein on platelets, HC platelets were preincubated with S or E protein for 5 min at 37? before incubated with PE-CD62P antibodies or AF647-Fg. To examine the effect of SB203580 or FA6-152 on E protein-enhanced platelet activation, HC platelets were pretreated with SB203580 or FA6-152 for 10 min at 37? before incubation with the E protein.
Instrument	FACS CantoII, Becton Dickinson
Software	BD FACSDiva software (version 8.0.1); FlowJo V10.4.0
Cell population abundance	Platelets were gated according to FSC-A/SSC-A and singlets were selected by FSC-A/FSC-W followed by CD41-positive and CD45-negative confirmation. The purity of the platelets was more than 95% detected by flow cytometry.
Gating strategy	Platelets were gated according to FSC-A/SSC-A and singlets were selected by FSC-A/FSC-W. Positive expression of Fg bindig and P-selectin exposure were determined by comparing the stained platelets with non-stained controls. MFI of stained platelets was evaluated.

☒ Tick this box to confirm that a figure exemplifying the gating strategy is provided in the Supplementary Information.
