# Supplementary material for: CD36 mediates SARS-CoV-2-envelope-protein-induced platelet activation and thrombosis
Source: Nat Commun. 2023 Aug 21;14:5077. doi: 10.1038/s41467-023-40824-7 (PMC10442425; doi:10.1038/s41467-023-40824-7)
Supplement: Supplementary file 2 — Peer Review File [file 41467_2023_40824_MOESM2_ESM.pdf]

nature portfolio

Peer Review File

CD36 mediates SARS-CoV-2-Envelope-protein-induced platelet activation and thrombosis

Open Access This file is licensed under a Creative Commons Attribution 4.0 International License, which permits use, sharing, adaptation, distribution and reproduction in any medium or format, as long as you give appropriate credit to the original author(s) and the source, provide a link to the Creative Commons license, and indicate if changes were made. In the cases where the authors are anonymous, such as is the case for the reports of anonymous peer reviewers, author attribution should be to 'Anonymous Referee' followed by a clear attribution to the source work. The images or other third party material in this file are included in the article's Creative Commons license, unless indicated otherwise in a credit line to the material. If material is not included in the article's Creative Commons license and your intended use is not permitted by statutory regulation or exceeds the permitted use, you will need to obtain permission directly from the copyright holder. To view a copy of this license, visit http://creativecommons.org/licenses/by/4.0/.

REVIEWER COMMENTS

Reviewer #1 (Remarks to the Author):

This is an interesting manuscript, by Tang and colleagues, which examines a novel mechanism behind thrombosis and platelet activation in COVID-19 patients. The mechanism proposed involves the SARS-CoV-2 envelope (E) protein that binds CD36 on platelets and mediates hyperactivation of human and mouse platelets through the p38 MAPK-NF-κB signaling pathway. Experiments in mice with genetic deficiency of CD36 supported the role of CD36.

Major concerns:

1. Human study - Did the authors also evaluate the relationship between serum E protein levels and biomarkers of platelet activation and aggregation?

2. Extended data Table 1 - other clinical information is needed, for example, the use of antiaggregants or metabolic profile parameters. Previous studies indicate that COVID-19 infection is associated with dyslipidemia with increased levels of OxLDL and higher levels in severely ill patients (doi:https://doi.org/10.1016/j.atherosclerosis.2022.06.496, doi.org/10.1155/2022/8067857). This information is of importance considering that OxLDL stimulates platelets through the ligation of CD36. Please add and discuss.

3. How were the concentrations to be used in vitro chosen? In some experiments, 0.5 μg/ml of E protein seems to be effective in platelet aggregation or in P-selectin expression (Figure 2). Did the authors test also lower concentrations (250 ng/ml for example)? Moreover, in Figure 3 they tested only the highest concentration (2 μg/ml). Is the only one working in these experiments? I think that using the lowest concentration corresponding to a concentration of E protein effectively measured in the patient’s serum can be more representative of the effect of E protein in the population analyzed. Indeed, for the mice model, the effective concentration is 4 μg corresponding to about 240 ng/ml.

4. In vitro, what is the effect of other agonists (collagen or thrombin) on platelet aggregation in the presence of E protein? Why authors tested only ADP?

5. S protein was used as a positive control in the experiments. Did the authors also evaluate the combined effect of the E protein and the S protein on platelet function?

6. The authors tested the inhibitory effect of SB203580 using E protein 0.5-2 μg/ml but the comparison reported is versus the activation with only E protein 2 μg/ml. Please add also the other 2 concentrations to the graph.

7. E protein also binds TLR2 which is expressed on platelets. What is the contribution of this receptor in this mechanism of activation? Indeed, in the presence of FA6-152, platelet function is ameliorated but

values do not return to the baseline. For platelet aggregation (Figure 5 panel d), the difference would seem significant.

8. Verification of CD36 and E protein interaction is critical. The authors should demonstrate E protein co-localization with CD36 by immunofluorescent staining or immunoprecipitation analysis in platelets.

9. CD36 expression on platelets varies widely (PMID: 21478428); moreover, a condition such as hyperglycemia is an important modulator of CD36 mRNA and protein expression. The authors should evaluate the CD36 expression and its p38 MAPK-NF-κB signaling pathway in COVID-19 compared to healthy subjects' platelets. In this regard, the description of the characteristics of the analyzed patients is also necessary as in point 2.

10. Extended data figure 4 – The expression of CD36 is heterogeneous on vascular endothelial cells. HUVEC expresses almost no CD36, whereas it is expressed in human pulmonary microvascular endothelial cells. The conclusion that E protein could activate platelet but not endothelial cells is not supported by the results.

11. Statistic methods should be better described, specifically how tests were used to analyze data.

Reviewer #2 (Remarks to the Author):

Tang et al present a very well-written and well-presented manuscript that convincingly demonstrates through a combination of a clinical cohort study and extensive experiments in vivo and in vitro that the SARS-CoV-2 E protein binds the membrane protein CD36 and activates platelet activation and thrombosis via a p38 MAPK and NFKB dependent pathway. Given the important role that plalelet activation and thrombosis have in severe COVID-19, these findings have important implications and to the best of my knowledge are novel.

The conclusions of the manuscript were well-supported by the data presented and I did not have any notable concerns regarding the methodology or analysis presented.

I recommend publication in Nature Communications.

Re: manuscript reference MS ID#: NCOMMS-23-09426-T #R1

REVIEWER COMMENTS

Reviewer #1 (Remarks to the Author):

This is an interesting manuscript, by Tang and colleagues, which examines a novel mechanism behind thrombosis and platelet activation in COVID-19 patients. The mechanism proposed involves the SARS-CoV-2 envelope (E) protein that binds CD36 on platelets and mediates hyperactivation of human and mouse platelets through the p38 MAPK-NF-κB signaling pathway. Experiments in mice with genetic deficiency of CD36 supported the role of CD36.

Response: We thank the reviewer for all the positive comments on our manuscript and the constructive suggestions on improving our manuscript.

Major concerns:

1. Human study - Did the authors also evaluate the relationship between serum E protein levels and biomarkers of platelet activation and aggregation?

Response: CD62P (P-selectin) is a granule-associated glycoprotein rapidly secreted from platelets in response to various physiological or pathological stimuli. Therefore, plasma soluble CD62P is a common biomarker of platelet activation. We enrolled another cohort of 91 COVID-19 patients to evaluate the relationship between serum E protein levels and CD62P levels. All included patients had not taken any antiplatelet medications in the one month prior to enrollment. Specifically, we collected plasma samples to measure CD62P levels and corresponding serum samples to measure E protein levels using ELISA assays. Among these patients, 40 had detectable levels of E protein and all the enrolled patients had detectable levels of CD62P. A Spearman correlation analysis showed a significant positive correlation between E protein levels and CD62P levels in this cohort of COVID-19 patients ($r=0.2066$, $P=0.0494$). We have included the data in the **Supplementary Figure 1f** and provided corresponding explanations in the revised **Results (Page 5, lines 116-117)**.

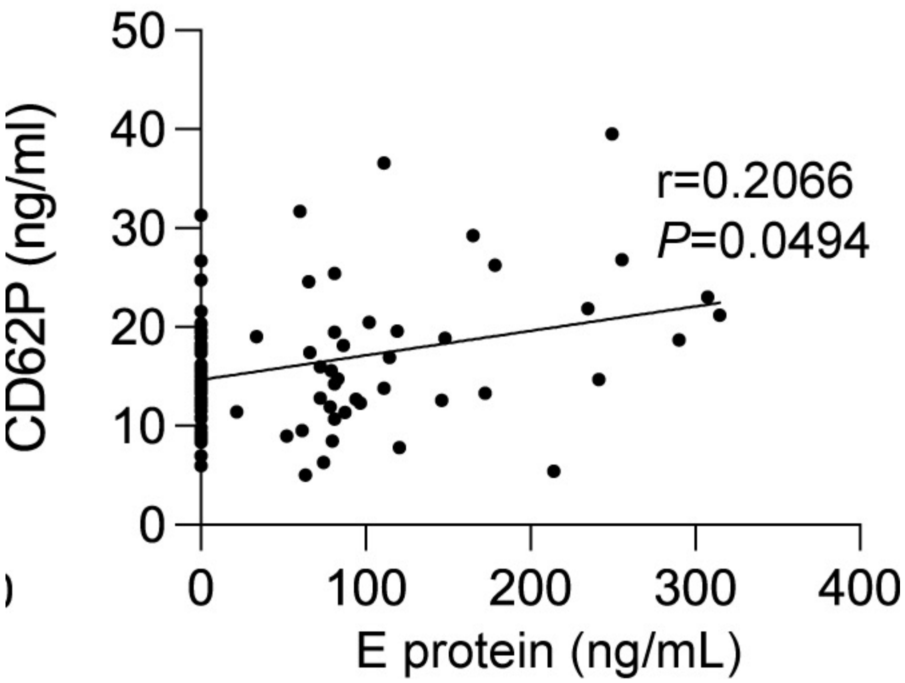

Supplementary Figure 1f. Correlation between E protein levels and CD62P levels (r=0.2066, *P*=0.0494; n=91).

2. Extended data Table 1 - other clinical information is needed, for example, the use of antiaggregants or metabolic profile parameters. Previous studies indicate that COVID-19 infection is associated with dyslipidemia with increased levels of OxLDL and higher levels in severely ill patients (doi:https://doi.org/10.1016/j.atherosclerosis.2022.06.496, doi.org/10.1155/2022/8067857). This information is of importance considering that OxLDL stmulates platelets through the ligation of CD36. Please add and discuss.

Response 2.1： We sincerely appreciate the constructive suggestion provided by the reviewer on this matter. We have supplemented the treatment plans for the patients enrolled during their hospitalization: a group of 28 individuals with concurrent coronary heart disease or cerebral infarction were administered antiplatelet medications. Out of these, 12 patients were prescribed aspirin and 16 patients received clopidogrel as antiaggregant regimen. These data have been incorporated into **Supplementary Table 1**.

Response 2.2： We thank our reviewer for bringing up the attention to increased levels of OxLDL post COVID-19 infection. Indeed, COVID-19 has been associated with

alterations in lipid profiles. Studies have shown that COVID-19 patients may experience changes in blood lipid levels, including increased levels of triglycerides and low-density lipoprotein cholesterol (LDL-C). Elevated LDL-C levels have been observed in severe cases and may be linked to the inflammatory response and liver dysfunction commonly seen in these patients[1,2]. Moreover, it has been reported that dysregulated platelet lipid metabolism plays a role in the occurrence of thrombotic complications in COVID-19[3].

In our new COVID-19 cohort of 91 patients, 40 patients presented with E protein. We thus conducted an analysis of the blood glucose and lipid profiles in these 40 patients. The results revealed the following (presented as mean ± SD): triglycerides 1.35 ± 0.59, cholesterol 3.87 ± 1.07, LDL 2.32 ± 0.88, HDL 1.03 ± 0.24, and blood glucose 5.52 ± 0.95. Notably, all of these values fell within the normal ranges. Considering that CD36 is a key receptor molecule involved in the cellular regulation of lipid influx, we acknowledge the significance of this issue and future studies are warranted to explore the role of E protein in the regulation of platelet lipid metabolism to investigate this matter further[4,5]. And we have commented on these issues in revised **Discussion (Pages 10-11, lines 292-298)**.

3. How were the concentrations to be used in vitro chosen? In some experiments, 0.5 μg/ml of E protein seems to be effective in platelet aggregation or in P-selectin expression (Figure 2). Did the authors test also lower concentrations (250 ng/ml for example)? Moreover, in Figure 3 they tested only the highest concentration (2 μg/ml). Is the only one working in these experiments? I think that using the lowest concentration corresponding to a concentration of E protein effectively measured in the patient's serum can be more representative of the effect of E protein in the population analyzed. Indeed, for the mice model, the effective concentration is 4 μg corresponding to about 240 ng/ml.

Response： We acknowledge the reviewers' concern regarding concentrations of E protein to be used *in vitro* and *in vivo* experiments. And, indeed, 4 μg of E protein per

mouse is estimated to be equivalent to 240 ng/ml in the sera of humans (according to the dose translation between laboratory animals and human in drug development). We also agreed with the reviewer that, in an ideal setup, "using the lowest concentration corresponding to a concentration of E protein effectively measured in the patient's serum".

Nevertheless, it is our opinion that the E protein concentration detected in patient sera likely corresponded to an "equilibrium" level of the concentration of the E protein, and the time delay between sample collection and experimental measurement could have resulted in some degrees of degradation of the protein. Therefore, the observed values could be an underestimate of the "real" concentration of E proteins in patients. Given this complication, the doses for in vitro and in vivo experiments in our study were mainly empirically determined by preliminary dose-escalation experiments. As shown in the manuscript, dose-escalation experiments (0.5/1/2 μg/ml) were performed for most of the in vitro experiments (Figures 3&4). While 0.5 μg/ml induced did not elicit statistically significant effect in some experiments (e.g., platelet aggregation, p=0.0203, Fig.3a; P-selectin MFI, p=0.0209, Fig. 3b), while significant in others (e.g., Fg binding MFI, p=0.0165, Fig.3c; spreading area, p=0.0001, Fig.3d; clot retraction, p=0.0001). Based on these observations, we thought that 0.5 μg/ml was likely close to, or even below, the threshold required to consistently induce platelet activation phenotypes and no lower concentrations (e.g., 250 ng/ml) were attempted subsequently.

On the other hand, as the reviewer pointed out, only the highest concentration (2 μg/ml) were used for the RNA-seq experiment shown in Figure 4a. The rationale for selecting this concentration as based on the results shown in Figure 3, since 2 μg/ml showed consistently larger and more significant effect on platelet activation phenotypes.

4. In vitro, what is the effect of other agonists (collagen or thrombin) on platelet aggregation in the presence of E protein? Why authors tested only ADP?

Response: E protein exhibits the ability to enhance platelet activation in the presence of collagen, thrombin, and ADP (**Fig. 1 for reviewers**). Notably, collagen and thrombin

act as strong platelet agonists, capable of activating platelets to the upper threshold level even at low concentrations. In contrast, ADP functions as a moderate-strength stimulant, enabling us to observe the augmented effect of E protein on platelet activation specifically in the presence of ADP.

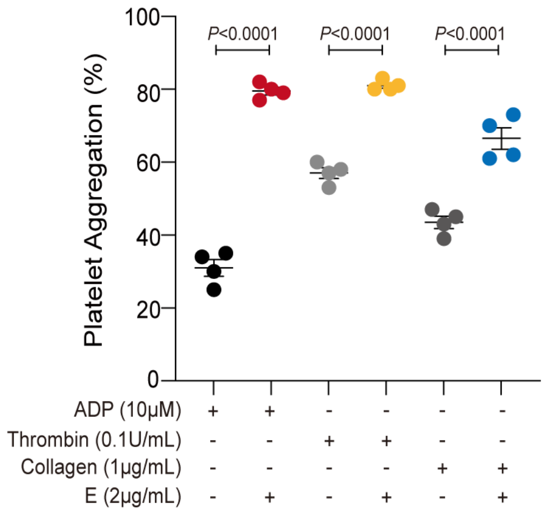

Fig. 1 for reviewers. Aggregation of pretreated platelets with the E protein (2μg/ml) was measured in response to ADP (10μM), thrombin (0.1U/mL), collagen (1μg/mL) (n=4). Data were analyzed by 1-way ANOVA with Tukey multiple-comparisons test. Data are presented as mean±SEM.

5. S protein was used as a positive control in the experiments. Did the authors also evaluate the combined effect of the E protein and the S protein on platelet function?

Response: We evaluate the combined effect of the E protein and the S protein on platelet spreading. Interestingly, E and S, at intermediate stimulation concentrations (E protein at 1μg/ml and S protein at 2μg/ml, respectively), can synergistically enhance platelet activation, indicating that they have distinct mechanisms for platelet activation (**Fig. 2 for reviewers**).

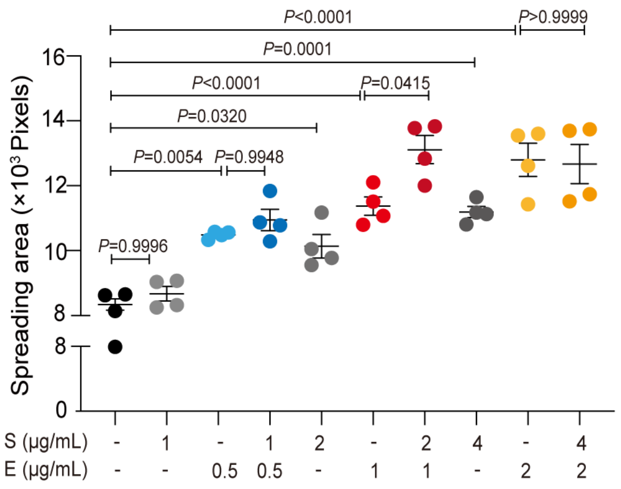

Fig. 2 for reviewers. Platelets pre-treated with different concentrations of E protein (0.5, 1, 2 µg/ml) and S protein (1, 2, 4 µg/ml), as well as the combination of E and S proteins, were allowed to spread on immobilized fibrinogen at 37°C for 40 minutes, followed by the measurement of spreading areas. Data were analyzed by 1-way ANOVA with Tukey multiple-comparisons test. Data are presented as mean±SEM.

6. The authors tested the inhibitory effect of SB203580 using E protein 0.5-2 µg/ml but the comparison reported is versus the activation with only E protein 2 µg/ml. Please add also the other 2 concentrations to the graph.

Response: We conducted an investigation into the inhibitory effect of SB203580 on E protein at various concentrations. As anticipated, the inhibitor demonstrated a higher relative inhibition efficiency for lower concentrations of E protein, compared to E protein at higher concentrations. Taking your suggestion into consideration, we have included additional data on the inhibitory effect of SB203580 on E protein stimulation at different concentrations (**Fig. 3 for reviewers**).

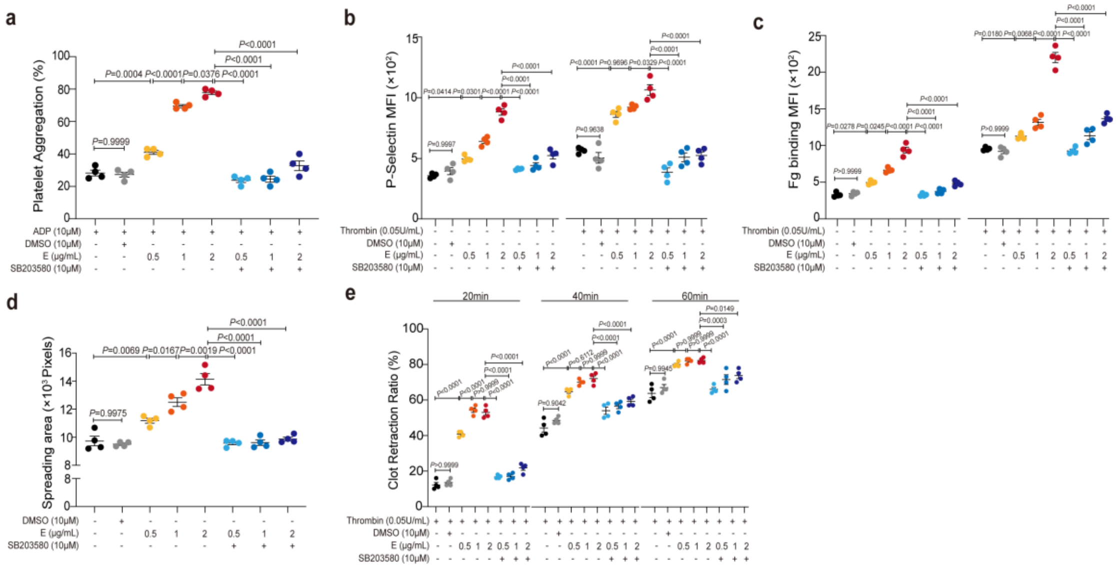

Fig. 3 for reviewers. (a) Potentiated platelet aggregation induced by the E protein combined with ADP 10μM was suppressed by SB203580 (n=4). (b, c) Enhanced P-selectin exposure and Fg binding of platelets induced by the E protein were suppressed by SB203580 (n=4). P-selectin exposure and binding of Alexa 647-Fg to pretreated platelets was measured in the presence or absence of thrombin (0.05U/ml). (d) SB203580 inhibited the E protein promoted human platelet spreading on Fg (n=4). Pretreated platelets were allowed to spread on fibrinogen-coated surface at 37°C for 1h. Data was showed with quantification analysis of the areas of spreading platelets (pixel numbers). (e) SB203580 attenuated accelerating clot retraction induced by the E protein in response to thrombin (n=4). Data was presented with quantification analysis of clot retraction at 20, 40 and 60 minutes. The E protein (0.5, 1, 2μg/ml), SB203580 (10μM), and DMSO (10μM) were used. Data were analyzed by 1-way ANOVA with Tukey multiple-comparisons test (a-d) or 2-way ANOVA with Tukey multiple-comparisons test (e). Data are presented as mean±SEM.

7. E protein also binds TLR2 which is expressed on platelets. What is the contribution of this receptor in this mechanism of activation? Indeed, in the presence of FA6-152, platelet function is ameliorated but values do not return to the baseline. For platelet aggregation (Figure 5 panel d), the difference would seem significant.

Response: Previous study has reported that E protein can bind to TLR2 on monocytes, leading to the release of inflammatory cytokines[6]. TLR2 is also expressed on platelets,

raising an intriguing question regarding the involvement of platelet TLR2 in E protein-mediated activation. In our *in vitro* experiments, we employed a blocking antibody against TLR2 during E protein stimulation. The results revealed that the TLR2 blocking antibody did not exert a significant inhibitory effect on the P-selectin expression and platelet spreading stimulated by E protein **(Fig. 4 for reviewers).** However, further investigations utilizing conditional TLR2 knockout and TLR2/CD36 double knockout mice can provide deeper insights into this matter.

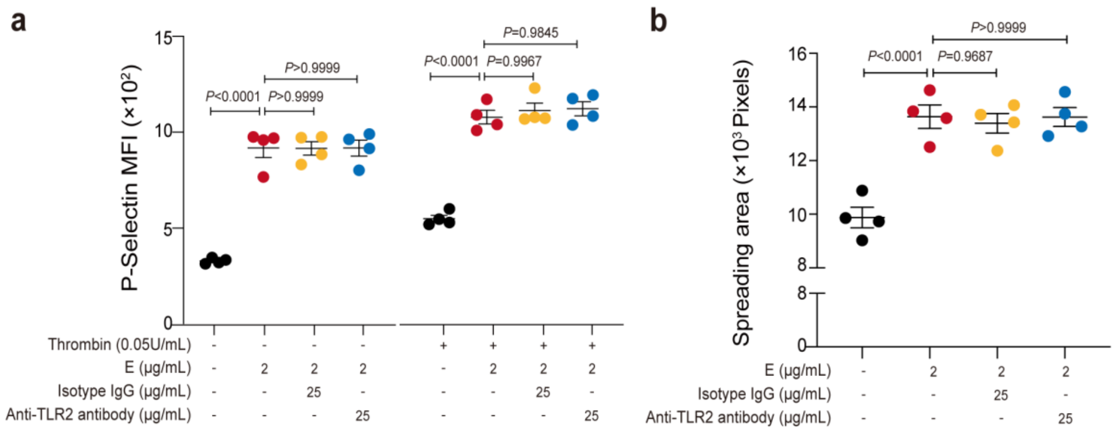

Fig. 4 for reviewers. (a) Human platelets were incubated with 25μg/ml anti-TLR2 antibody or 25μg/ml isotype control IgG for 10 minutes at 37℃, and then treated with the E protein 2μg/ml for 5 minutes. P-selectin exposure and binding of Alexa 647-Fg to platelets was measured with or without stimulation of thrombin (0.05U/ml). (b) After incubation with 25μg/ml anti-TLR2 antibody or 25μg/ml isotype control IgG for 10 minutes at 37℃, washed human platelets were treated with the E protein 2μg/ml for 5 minutes. Platelets were allowed to spread on immobilized fibrinogen at 37℃ for 1h. Area (pixel numbers) of 3 random fields of spreading platelets were quantified (n=4). Data were analyzed by 1-way ANOVA with Tukey multiple-comparisons test. Data are presented as mean±SEM.

8. Verification of CD36 and E protein interaction is critical. The authors should demonstrate E protein co-localization with CD36 by immunofluorescent staining or immunoprecipitation analysis in platelets.

Response: In addition to validating the binding of E protein and CD36 molecules through His-pull down assay and CO-IP assay, we further confirmed their interaction using Surface Plasmon Resonance experiments to assess the affinity between the E protein and human CD36. The E protein was able to bind to recombinant human CD36 with high affinity (K_D=55 nM). Interactions between the E protein and CD36 were observed in a dose-dependent manner (**Figure 5a**). Moreover, the colocalization of the E protein and CD36 were identified on platelets isolated from COVID-19 patients by immunofluorescent staining (**Supplementary Fig. 8**). As suggested, we have provided these data in **Result (Page 8, lines 207-211; 216-217)** and **Methods (Pages 15-16, lines 449-456; Page 18, lines 515-523).**

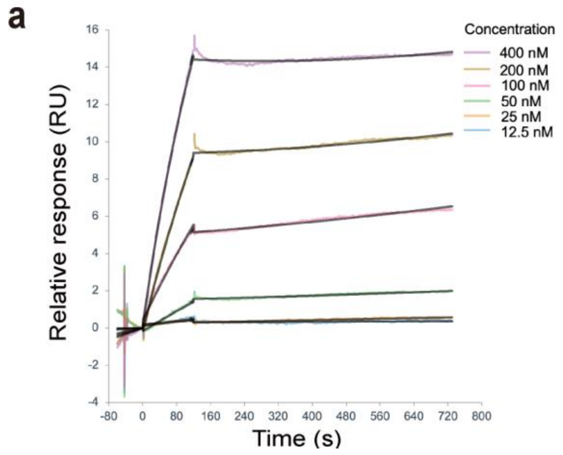

Figure 5a. Biacore sensorgrams and binding kinetics determined by SPR for recombinant SARS-CoV-2 E protein with recombinant human CD36 protein.

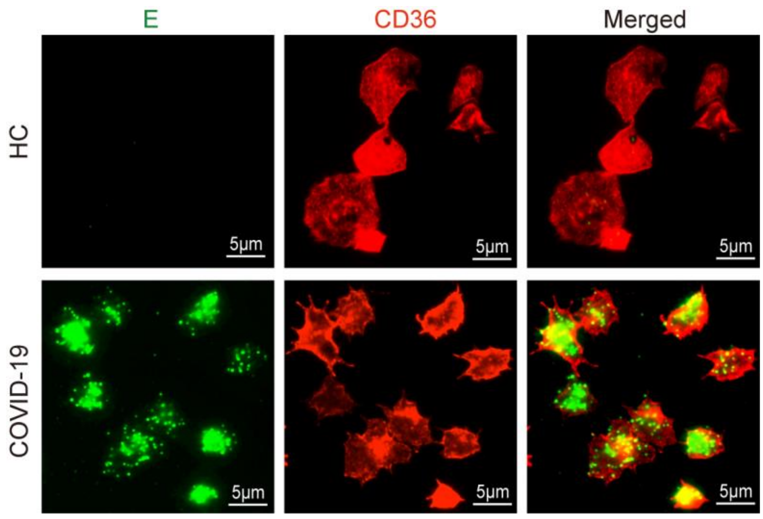

Supplementary Figure 8. The E protein and CD36 colocalized on platelets from COVID-19 patients. The colocalization of the E protein and CD36 on COVID-19 platelets. Immunofluorescence staining on platelets isolated from COVID-19 patients and HC was performed with anti-SARS-CoV-2 E protein (green), and anti-CD36 (red) antibodies. Scale bar=5μm.

9. CD36 expression on platelets varies widely (PMID: 21478428); moreover, a condition such as hyperglycemia is an important modulator of CD36 mRNA and protein expression. The authors should evaluate the CD36 expression and its p38 MAPK-NF-κB signaling pathway in COVID-19 compared to healthy subjects' platelets. In this regard, the description of the characteristics of the analyzed patients is also necessary as in point 2.

Response: Consistent with previous studies, COVID-19 platelets exhibited higher reactivity, characterized by increased aggregation capacity under ADP stimulation (**Supplementary Fig. 6a-b**), elevated P-selectin exposure (**Supplementary Fig. 6c**), increased spreading area on on immobilized fibrinogen (**Supplementary Fig. 6d-e**), and enhanced clot contraction **Supplementary Fig. 6f-g**). Of note, both the protein expression of CD36, p38 MAPK and NF-κB signaling pathways were significantly upregulated in COVID-19 platelets in the presence of agonists or not (**Supplementary Fig. 6h-k**). We have included this statement in the **Results (Page 7, Lines 179-186)**.

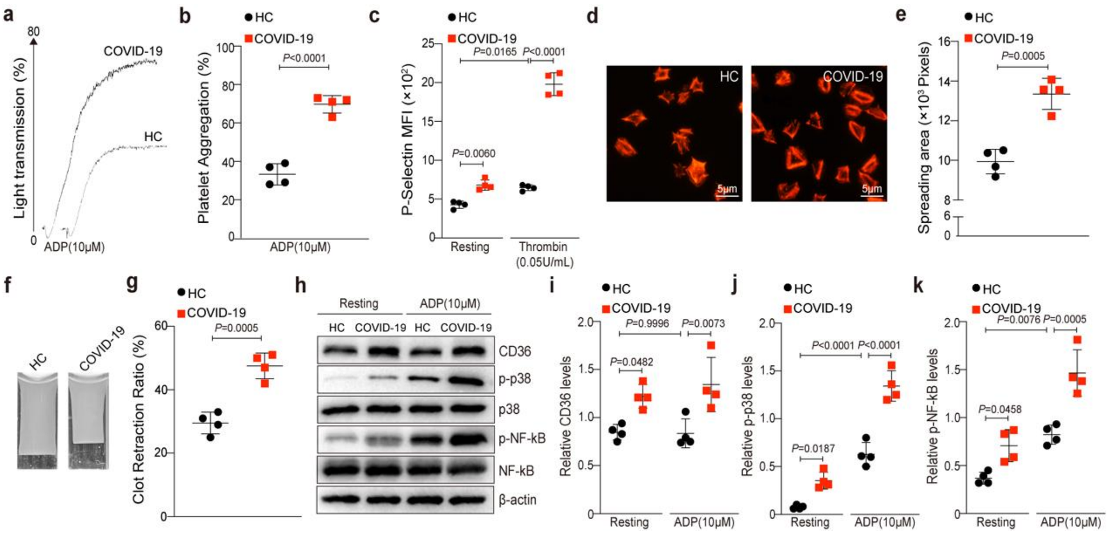

Supplementary Figure 6. Platelet activation is potentiated in COVID-19 patients.

a and **b** Aggregation of washed platelets isolated from COVID-19 patients and healthy donors (HC) in response to ADP (10μM) (n=4). **c** P-selectin exposure of platelets with or without stimulation of thrombin (0.05U/mL) (n=4). **d** and **e** Spreading of platelets from COVID-19 patients and HC on immobilized Fg (n=4). **f** and **g** Clot retraction of platelets from COVID-19 patients and HC induced by 1 U/ml thrombin was photographed and analyzed at 30 minutes (n=4). **h-k** The relative levels of CD36 and phosphorylation levels of p38 and NF-κB in human platelets isolated from COVID-19 patients and HC were detected by Western Blotting (n=4). Data were analyzed by unpaired Student *t* test (**b**, **e**, **g**), or 2-way ANOVA with Tukey multiple-comparisons test (**c**, **i-k**). Data are presented as mean±SD.

10. Extended data figure 4 – The expression of CD36 is heterogeneous on vascular endothelial cells. HUVEC expresses almost no CD36, whereas it is expressed in human pulmonary microvascular endothelial cells. The conclusion that E protein could activate platelet but not endothelial cells is not supported by the results.

Response: ICAM-1 (Intercellular Adhesion Molecule-1) and VCAM-1 (Vascular Cell Adhesion Molecule-1) are adhesion molecules that are primarily expressed on the endothelial cells of the inner surface of blood vessels. In this study, we employed E protein as a stimulant for endothelial cells and conducted Western blot (WB) analysis to assess the levels of ICAM-1 and VCAM-1. The findings revealed that E protein did not exert any significant influence on the protein levels of ICAM-1 and VCAM-1. Nevertheless, we acknowledge that this experiment alone may not provide definitive evidence to conclude that the E protein impacts on endothelial cell function. Further comprehensive research is warranted to delve into the potential effects of E protein on endothelial cell function， including vasodilation function testing, platelet adhesion testing, cell migration and invasion capability testing, and vascular permeability assay. And we add the following sentence in the revised **Results** to clarify this point (**Page 6, lines 158-160**).

"On the other hand, when incubated with endothelial cells, E protein did not seem to cause a change in the expression of ICAM-1 and VCAM-1 (Extended Data Fig.4a-c). This observation might suggest that the E protein did not notably activate endothelial

cells, though further functional studies on the impact of E protein on endothelial cells are required to address this.”

11. Statistic methods should be better described, specifically how tests were used to analyze data.

Response: We thoroughly reviewed and verified the statistical methods employed in each analysis throughout the study to ensure the accuracy of our data analysis. The following section in the Methods provides details on the statistical tests used in the study (**Page 18, Lines 524-532**). More specifically, we have clearly indicated the specific statistical analysis methods employed for each analysis in the figure legends.

Statistical analysis

Statistical analyses were performed using SPSS 26 (SPSS, Chicago, IL). Data were presented as the means±SEM or means±SD as indicated. Data were tested for normality and equal variance before parametric analysis. The Student (*t*) test, 1-way ANOVA, 2-way ANOVA, Kruskal-Wallis test, or Mann-Whitney *U* test were used, as appropriate. The statistical analysis method used for each experiment was indicated in the legend section. Graphs were generated using GraphPad Prism 6 Software. FlowJo V10.4.0 was used for flow cytometry analysis. All tests were 2-sided. P <0.05 was considered statistically significant.

Reviewer #2 (Remarks to the Author):

Tang et al present a very well-written and well-presented manuscript that convincingly demonstrates through a combination of a clinical cohort study and extensive experiments in vivo and in vitro that the SARS-CoV-2 E protein binds the membrane protein CD36 and activates platelet activation and thrombosis via a p38 MAPK and NFKB dependent pathway. Given the important role that platelet activation and thrombosis have in severe COVID-19, these findings have important implications and to the best of my knowledge are novel.

The conclusions of the manuscript were well-supported by the data presented and I did

not have any notable concerns regarding the methodology or analysis presented.

I recommend publication in Nature Communications.

Response： We are grateful for the time and effort for the reviewer in commenting on our work. And we thank the reviewer for all the positive comments on our manuscript.

Reference

1. Lalosevic M, Kotur-Stevuljevic J, Vekic J, et al. Alteration in Redox Status and Lipoprotein Profile in COVID-19 Patients with Mild, Moderate, and Severe Pneumonia. *Oxid Med Cell Longev.* 2022;2022:8067857.
2. Nakamura H, Kataoka Y, Nicholls SJ, et al. Elevated Lipoprotein(a) as a potential residual risk factor associated with lipid-rich coronary atheroma in patients with type 2 diabetes and coronary artery disease on statin treatment: Insights from the REASSURE-NIRS registry. *Atherosclerosis.* 2022;349:183-189.
3. Goracci L, Petito E, Di Veroli A, et al. A platelet lipidomics signature in patients with COVID-19. *Platelets.* 2023;34(1):2200847.
4. Qi Z, Hu L, Zhang J, et al. PCSK9 (Proprotein Convertase Subtilisin/Kexin 9) Enhances Platelet Activation, Thrombosis, and Myocardial Infarct Expansion by Binding to Platelet CD36. *Circulation.* 2021;143(1):45-61.
5. Chen Y, Zhang J, Cui W, Silverstein RL. CD36, a signaling receptor and fatty acid transporter that regulates immune cell metabolism and fate. *J Exp Med.* 2022;219(6).
6. Zheng M, Karki R, Williams EP, et al. TLR2 senses the SARS-CoV-2 envelope protein to produce inflammatory cytokines. *Nat Immunol.* 2021;22(7):829-838.

REVIEWERS' COMMENTS

Reviewer #1 (Remarks to the Author):

My concerns were completely addressed
